# LINC00571 drives tricarboxylic acid cycle metabolism in triple-negative breast cancer through HNRNPK/ILF2/IDH2 axis

**DOI:** 10.1186/s13046-024-02950-y

**Published:** 2024-01-18

**Authors:** Zihan Xi, Haohao Huang, Jin Hu, Yuanhang Yu, Xianxiong Ma, Ming Xu, Jie Ming, Lei Li, Hui Zhang, Hengyu Chen, Tao Huang

**Affiliations:** 1 Department of Breast and Thyroid Surgery, Union Hospital, Tongji Medical College, Huazhong University of Science and Technology, Wuhan, 430022 China; 2 Department of Neurosurgery, General Hospital of Central Theater Command of Chinese People’s Liberation Army, Wuhan, 430070 China; 3 General Hospital Of Central Theater Command and Hubei Key Laboratory of Central Nervous System Tumor and Intervention, Wuhan, China; 4 Department of Breast and Thyroid Surgery, Renmin Hospital of Wuhan University, Wuhan, 430060 China; 5 Cancer Center, Union Hospital, Tongji Medical College, Huazhong University of Science and Technology, Wuhan, 430022 China; 6 Department of Gastrointestinal Surgery, Union Hospital, Tongji Medical College, Huazhong University of Science and Technology, Wuhan, 430022 China; 7 Department of Urology, Union Hospital, Tongji Medical College, Huazhong University of Science and Technology, Wuhan, 430022 China; 8 Department of Breast and Thyroid Surgery, The Second Affiliated Hospital of Hainan Medical University, Haikou, 570216 China

**Keywords:** Triple-negative breast cancer, Tricarboxylic acid cycle, LINC00571, HNRNPK, ILF2, IDH2

## Abstract

**Background:**

Triple-negative breast cancer is a complex breast malignancy subtype characterized by poor prognosis. The pursuit of effective therapeutic approaches for this subtype is considerably challenging. Notably, recent research has illuminated the key role of the tricarboxylic acid cycle in cancer metabolism and the complex landscape of tumor development. Concurrently, an emerging body of evidence underscores the noteworthy role that long non-coding RNAs play in the trajectory of breast cancer development. Despite this growing recognition, the exploration of whether long non-coding RNAs can influence breast cancer progression by modulating the tricarboxylic acid cycle has been limited. Moreover, the underlying mechanisms orchestrating these interactions have not been identified.

**Methods:**

The expression levels of LINC00571 and IDH2 were determined through the analysis of the public TCGA dataset, transcriptome sequencing, qRT‒PCR, and Western blotting. The distribution of LINC00571 was assessed using RNA fluorescence in situ hybridization. Alterations in biological effects were evaluated using CCK-8, colony formation, EdU, cell cycle, and apoptosis assays and a tumor xenograft model. To elucidate the interaction between LINC00571, HNRNPK, and ILF2, RNA pull-down, mass spectrometry, coimmunoprecipitation, and RNA immunoprecipitation assays were performed. The impacts of LINC00571 and IDH2 on tricarboxylic acid cycle metabolites were investigated through measurements of the oxygen consumption rate and metabolite levels.

**Results:**

This study revealed the complex interactions between a novel long non-coding RNA (LINC00571) and tricarboxylic acid cycle metabolism. We validated the tumor-promoting role of LINC00571. Mechanistically, LINC00571 facilitated the interaction between HNRNPK and ILF2, leading to reduced ubiquitination and degradation of ILF2, thereby stabilizing its expression. Furthermore, ILF2 acted as a transcription factor to enhance the expression of its downstream target gene IDH2.

**Conclusions:**

Our study revealed that the LINC00571/HNRNPK/ILF2/IDH2 axis promoted the progression of triple-negative breast cancer by regulating tricarboxylic acid cycle metabolites. This discovery provides a novel theoretical foundation and new potential targets for the clinical treatment of triple-negative breast cancer.

**Supplementary Information:**

The online version contains supplementary material available at 10.1186/s13046-024-02950-y.

## Background

Breast cancer is one of the most prevalent malignancies among women worldwide [1, 2]. According to a 2022 statistical report, approximately 2.9 million new cases of breast cancer are diagnosed globally, constituting 31% of all malignant neoplasms in women [1]. Triple-negative breast cancer (TNBC), accounting for 10-20% of all cases of breast cancer [3], has a higher recurrence rate, poorer prognosis, and greater rate of malignancy than other subtypes [4]. Unfortunately, targeted and endocrine therapies have proven ineffective in TNBC patients [5]. Therefore, it has become crucial to explore the mechanism underlying these diseases to identify promising therapeutic targets.

Long non-coding RNAs (LncRNAs) are transcripts of more than 200 nucleotides, that have no protein coding potential [6]. Researchers have demonstrated the significant involvement of lncRNAs in the progression and metastasis of multiple malignant tumors [7–10]. LncRNAs exhibit diverse molecular mechanisms, such as acting as miRNA sponges [11], enhancing enhancer activity by physically binding to enhancer regions, binding with transcription factors to regulate gene expression [12], binding to antisense mRNAs to influence their post-transcriptional regulation [13], and interacting with one or more protein chaperones [14]. Furthermore, lncRNAs can act as scaffold molecules for the organization of macromolecular complexes [15]. These findings highlight the importance of lncRNAs in cancer research and underscore their potential as therapeutic targets.

RNA-binding proteins (RBPs) are involved in a wide variety of cellular processes, including gene expression regulation [16–19]. Heterogeneous nuclear ribonucleoprotein K (HNRNPK) is a vital member of the HNRNP family that functions as an RNA and DNA binding protein and engages in complex interactions with molecular partners to participate in chromatin remodeling, transcription, mRNA splicing, export, and translation processes [20–23]. Previous work has demonstrated that HNRNPK interacts with transcription factors or transcriptional repressor proteins involved in transcription, including p53, YBX1, and Zik1 [20, 24, 25]. Despite some progress, the molecular partners and functional mechanisms of HNRNPK in TNBC have yet to be thoroughly explored.

The tricarboxylic acid (TCA) cycle is a central hub for cellular energy metabolism, macromolecular synthesis, and redox balance. Recent studies have provided profound insights into the pivotal role of the TCA cycle in cancer metabolism, suggesting that it is a promising focal point for cancer therapy [26–28]. IDH2 is an essential rate-limiting enzyme in the TCA cycle that utilizes NADP+ mediators to convert isocitrate and α-ketoglutarate (α-KG) [29]. IDH2 exerts a significant influence on the progression of multiple cancers, including breast cancer [30–33]. However, the mechanism regulating the transcription of IDH2 has not been elucidated. It is imperative to explore the regulation of the TCA cycle and its rate-limiting enzymes.

In the present study, we revealed that the expression level of LINC00571 was markedly upregulated in TNBC samples and that LINC00571 facilitated the progression of TNBC. Specifically, LINC00571 acted as a scaffold, facilitating the interaction between HNRNPK and ILF2, thereby regulating the transcription of IDH2 and promoting the TCA cycle. Consequently, targeting this mechanism holds is a promising potential therapeutic strategy for TNBC.

## Methods

### Patients and specimens

Tumor and paracancerous tissues were obtained from TNBC patients who had not undergone chemotherapy or radiotherapy during surgery at Union Hospital, Tongji Medical College, Huazhong University of Science and Technology, and demographic and clinicopathological information was collected concurrently. The Institutional Review Board of Tongji Medical College approved the human tissue study. All procedures were implemented in accordance with the 'Helsinki Declaration' rules. Written informed consent was obtained from all patients. The tumor tissues were confirmed as TNBC by pathological diagnosis, and the tissues were frozen in liquid nitrogen and stored at -80 °C.

### Cell culture and reagents

The human normal mammary epithelial cell line (MCF10A) and TNBC cell lines MDA-MB-231 and BT-549 were purchased from the American Type Culture Collection (ATCC, USA). MCF10A cells were cultured in DMEM/F12 medium (Gibco, USA) supplemented with 5% horse serum (Gibco, USA), 20 ng/ml recombinant epidermal growth factor (PeproTech, USA), 0.5 μg/ml hydrocortisone (Sigma, USA), 10 μg/ml insulin (Sigma, USA), 0.1 μg/ml cholera toxin (Sigma, USA), and 1% antibiotics (Invitrogen, USA). MDA-MB-231 cells were cultured in DMEM medium (GIBCO, USA), and BT-549 cells were cultured in RPMI 1640 medium (GIBCO, USA) supplemented with 10% fetal bovine serum (GIBCO, USA). All cells used in this study were cultured in a 5% CO2 incubator at 37 °C.

### RNA extraction and quantitative real-time PCR analysis

Total RNA was extracted from cell lines or fresh tissues using TRIzol reagent (Invitrogen, USA). cDNA was synthesized using a PrimeScript RT kit (TaKaRa, Japan). qRT‒PCR was performed using the StepOnePlus Real-Time PCR System (Applied Biosystems, USA). A StepOnePlus Real-Time PCR System (Applied Biosystems, USA) was used for qRT‒PCR. The mRNA internal controls used were GAPDH and U6. Relative RNA abundance was calculated using the standard 2-ΔΔCt method. Relative RNA abundance was calculated using the standard 2-ΔΔCt method with the following formula: ∆Ct = Ct (gene of interest) – Ct (gene of internal controls), ∆∆Ct = ∆Ct (sample) – ∆Ct (control average), and fold gene expression = 2^-(∆∆Ct). On the resulting bar chart, the y-axis shows the fold change in mRNA expression relative to the control. The primers used in the study were synthesized by TSINGKE Biotech (Beijing, China) and are shown in Additional file 13.

### Subcellular fractionation

Nuclear and cytoplasmic separation were performed using a PARIS Kit (Life Technologies, USA) according to the manufacturer’s instructions.

### RNA fluorescence in situ hybridization

RNA fluorescence in situ hybridization (FISH) was performed on TNBC cell lines using a Fluorescence In Situ Hybridization Kit (RiboBio, China). Antisense and sense probes containing the LINC00571 linker sequence were synthesized. The cells were seeded on confocal dishes. Cells were fixed in 4% paraformaldehyde (Thermo Scientific, Rockford, IL, USA) for 15 minutes, washed three times for 5 minutes per wash with PBS (Sigma, Aldrich, USA), incubated with 0.5% Triton X-100/PBS osmotic solution at room temperature for 5-10 minutes, and then washed three times with PBS for 5 minutes per wash. The cells were incubated with the specific probe at 37 °C overnight. Cell nuclei were stained with DAPI. Fluorescence images were captured using a Nikon A1R-si laser scanning confocal microscope (Nikon, Japan). The probe sequence used for FISH is shown in Additional file 13.

### Plasmid construction and stable transfection

Short hairpin RNAs targeting LINC00571, HNRNPK, ILF2, and IDH2 (Additional file 13) were synthesized by TSINGKE and cloned into pLKO.1-puro (Sigma‒Aldrich). Human cDNAs for LINC00571, HNRNPK, ILF2, and IDH2 were synthesized by TSINGKE (Wuhan, China) and cloned into pLVX-puro (Takara Bio, Japan) and p3XFLAG-CMV-10 (Sigma-Aldrich) vectors to construct overexpression plasmids. Truncated HNRNPK was amplified using specific primers (Additional file 13) and subcloned into the p3XFLAG CMV-10 vector. The plasmids were transfected into breast cancer cells by using Lipofectamine 3000 (Invitrogen, USA) according to the manufacturer’s instructions. Stable cell lines were screened using neomycin or puromycin (Invitrogen, USA). Interfering shRNA (shNC) and empty vector were utilized as controls (Additional file 13).

### CCK8 assay

A Cell Counting Kit-8 (CCK8) assay was performed according to the manufacturer’s protocol to evaluate the proliferation efficiency of the TNBC cells. At a density of 5000 cells/well, TNBC cells were seeded into 96-well plates. Then, 10% CCK-8 solution (Dojindo, Japan) was added to the 96-well plates, which were incubated in a 37 °C incubator for 1 hour. The spectral absorbance of each well at 450 nm was measured on a microplate reader (Thermo Fisher, USA).

### Colony formation assay

After relevant transfection with the relevant plasmids, TNBC cells (1000 cells/well) were seeded into 6-well plates and incubated at 37 °C. After approximately two weeks, the cell colonies were washed with PBS, fixed with 4% paraformaldehyde (Thermo Scientific, USA) for 10 minutes, stained with 0.2% crystal violet (Solarbio, China) for 15 minutes, imaged and counted.

### EdU proliferation assay

The assays were performed with an EdU incorporation assay kit (RiboBio, China) according to the manufacturer’s instructions. Transfected cells (2 × 10^4^ cells/well) were seeded into 96-well plates. After 24 hours, 100 μl of medium containing 50 mM EdU was added to each well, and the cells were then incubated for 2 hours at 37 °C. The cells were then fixed with 4% paraformaldehyde and stained with Hoechst and Apollo reaction cocktail.

### Flow cytometry analysis

An Annexin V-FITC/PI Apoptosis Detection Kit (Vazyme, China) was used to evaluate cell apoptosis. The cells were harvested and washed twice with 4 °C precooled PBS. The cell precipitates were resuspended in 200 µL of 1× binding buffer, stained with 5 µL of Annexin V-FITC/PI, and protected from light for 15 minutes at room temperature. PI/RNase Staining Buffer (BD Biosciences, USA) was used to assess the cell cycle distribution. The cells were collected and washed with precooled PBS at 4 °C. The cell precipitates were fixed overnight at -20 °C in 75% ethanol, washed twice with pre-cooled PBS at 4 °C, stained with 200 µl of PI/RNase Staining Buffer, and protected from light for 15 minutes at room temperature. Apoptosis and cell cycle stage were valuated via flow cytometry and analyzed via FlowJo software.

### Western blot analysis

The proteins were extracted and separated via 10% SDS‒PAGE gel and transferred to 0.22 µm PVDF membranes (Millipore, USA). The membrane was blocked with 5% skim milk powder and incubated with specific antibodies at 4 °C overnight. The membrane was then incubated with the appropriate secondary antibody, and protein bands were detected using an enhanced chemiluminescence (ECL) detection system (Bio-Rad, USA), and images were acquired using a Bio Spectrum 600 imaging system (UVP). GAPDH was used as a control. The antibodies used included primary antibodies against HNRNPK (11426-1-AP, Proteintech, China), ILF2 (ab113205, Abcam, UK), ILF2 (67685-1-Ig, Proteintech, China), GAPDH (60004-1-Ig, Proteintech, China), IDH2 (15932-1-AP, Proteintech, China), Flag (AE005, ABclonal, China), ubiquitin (10201-2-AP, Proteintech, China). HRP-conjugated secondary goat anti-mouse (SA00001-1, Proteintech, China), and goat anti-rabbit (SA00001-2, Proteintech, China).

### Tumor xenograft model

All animal experiments were performed in accordance with the NIH Guide for the Care and Use of Laboratory Animals, and approved by the Animal Care Committee of Tongji Medical College. For subcutaneous xenograft tumor models, MDA-MB-231 cells (5×10^6^) were injected into the upper back of 4-week-old female BALB/c nude mice (*n*=5 per group). For orthotopic breast tumors models, 3 × 10^6^ MDA-MB-231 cells were injected into the mammary fat pad of 8-week-old female BALB/c nude mice (*n*=5 per group). Tumor sizes were measured using a caliper, and tumor volume was evaluated = length × (width^2)/2.

### Immunohistochemistry

Immunohistochemical staining and quantitative evaluation were carried out with antibodies specific for Ki-67 (GB111499, Servicebio, China) and PCNA (GB11010; Servicebio, China). According to the percentage of positive cancer cells, the degree of positivity was measured.

### TUNEL assay

TUNEL staining was performed on xenograft tumor tissue according to the guidelines of the TUNEL Apoptosis Detection Kit (Vazyme, China). Images were captured using an Olympus FSX100 microscope (Hungary).

### Measurement of oxygen consumption rate

The oxygen consumption rate (OCR) was measured using an XFe96 Extracellular Flux Analyzer (Seahorse Bioscience, Agilent). A total of 2 ×10^4^ cells were seeded in XFe96 cell culture microplates precoated with 20 mg/mL poly-Dlysine, to reach 90% confluence before measurements. The medium was replaced with XF base medium (Seahorse Bioscience), and the cells were incubated at 37 ℃ without CO_2_ for 45 minutes prior to the assay. The OCR was measured under basal respiration, upon sequential treatment with 1 μM oligomycin, 0.5 μM carbonyl cyanide-4-(trifluoromethoxy) phenylhydrazone (FCCP), and 1 μM rotenone plus antimycin A. The OCR was normalized to the cell index as measured by crystal violet staining at the end of the experiment.

### Lactate level detection

A total of 5 × 10^6^ cells were harvested and the lactate content was determined according to the instructions of the Lactate Content Assay Kit (Solarbio, BC2230, China). To construct a standard curve, the concentration of each standard solution was used as the x-axis, and the corresponding absorbance values (ΔA standard) served as the y-axis. The standard curve was then used to obtain the standard equation, y=kx+b. We subsequently calculated the lactate content by entering the ΔA measurement values into the following equation: lactate content (μmol/10^6^ cells) = 1.1875 × [(ΔA measurement – b)/k]/ cell count.

### ATP measurement

The culture medium was first aspirated, and subsequently, 5 x 10^6^ cells were collected. The ATP concentration within the cells was detected using an ATP Content Assay Kit (Solarbio, BC0305, China). The ATP content (μmol/10^6^ cells) was calculated with the following formula: ATP content (μmol/10^6^ cells) = 0.125 × ΔA (sample)/ ΔA (standard). In this equation, ΔA (sample) represents the change in absorbance measured for the sample, while ΔA (standard) denotes the change in absorbance measured for the standard.

### Citrate and α-KG level detection

A total of 5 x 10^6^ cells were harvested and the concentration. of citrate was determined using a Citric Acid Content Assay Kit (Solarbio, BC2155, China). The citrate content (μmol/10^4^ cells) was calculated as follows: citrate content (μmol/10^4^ cells) = 2 × ΔA (sample - blank)/ ΔA (standard - blank)/ cell count. Similarly, the α-ketoglutarate (α-KG) content was measured using a α-Ketoglutaric Acid (α-KG) Content Assay Kit (Solarbio, BC5425, China) according to the following formula: α-KG content (nmol/10^6^ cells) = 475 × ΔA (sample - blank)/ ΔA (standard - blank)/ cell count × sample dilution factor.

### RNA pull-down and mass spectrometry

Full-length LINC00571 was synthesized by in vitro transcription using the T7 High Yield RNA Transcription Kit (Vazyme, China) and labelled with biotin-14-dCTP according to the manufacturer's protocol (Invitrogen, Grand Island, NY, USA). TNBC cells (2 x 10^7^) were harvested and lysed to extract protein. The lysates, biotin-labelled RNA and streptavidin C1 magnetic beads (Invitrogen, USA) were incubated for 12 hours at 4 °C, after which the beads were washed four times with lysis buffer. Purified nuclear proteins were analysed by boiling the RNA‒protein binding mixture in SDS buffer followed by mass spectrometry.

### Silver staining and mass spectrometry analysis

Silver staining was performed using a PAGE Gel Silver Staining Kit (Solarbio, China) according to the manufacturer’s protocol, after which a mass spectrometry analysis was performed to analyze the purified nuclear proteins. Liquid chromatography‒mass spectrometry (LC‒MS) (Novogene, China) was used for mass spectrometry analysis.

### RNA immunoprecipitation assay

RNA immunoprecipitation (RIP) assays were performed according to the instructions of the Thermo Scientific RIP Kit (Thermo, Waltham, MA, USA) using specific antibodies against HNRNPK (11426-1-AP, Proteintech, China), ILF2 (ab113205, Abcam, UK), Flag (ab45766, Abcam, UK), or IgG (30000-0-AP, Proteintech, China) and Protein A/G magnetic beads (Life Technologies, USA) for RNA immunoprecipitation. Input and coprecipitated RNA were detected via qRT‒PCR analysis.

### Immunofluorescence analysis

Cells were seeded on confocal dishes. The cells were fixed in 4% paraformaldehyde (Thermo Scientific, Rockford, IL, USA) for 15 minutes, washed three times with PBS (Sigma‒Aldrich, USA), incubated with 0.5% Triton X-100/PBS at room temperature for 5-10 minutes, and then washed three times with PBS. The cells were blocked with 1% BSA for 30 minutes at 37 °C and then incubated with HNRNPK antibody at 4 °C overnight. On the second day, the cells were washed with PBS and incubated with an anti-ILF2 antibody overnight at 4 °C. On the third day, the cells were washed with PBS and incubated with the appropriate secondary antibody at 37 °C for 30 minutes. Cell nuclei were stained with DAPI. Fluorescence images were captured using a Nikon A1R-si laser scanning confocal microscope (Nikon, Japan).

### Coimmunoprecipitation assay

A total of 10^7^ cells were subjected to cell lysis by NP40 supplemented with protease inhibitors for 30 minutes on ice. The cell lysates were incubated with protein A/G agarose (Thermo Fisher Scientific, #20421, USA) and antibodies or IgG at 4 °C overnight. The cell lysates were then washed three times with PBST buffer and eluted at 95 °C for 10 minutes. The eluted proteins were detected by Western blotting. The following antibodies were used for coimmunoprecipitation (Co-IP) analysis: HNRNPK (11426-1-AP, Proteintech, China), ILF2 (ab113205, Abcam, UK), and IgG (30000-0-AP, Proteintech, China).

### Luciferase reporter

PGL3-basic luciferase reporter plasmids were transfected into breast cancer cells by using Lipofectamine 3000 (Invitrogen, USA) according to the manufacturer’s instructions. After 24-36 hours of transfection, the cells were lysed, and firefly luciferase and Renilla luciferase activities were measured using a Luciferase Reporter Gene Assay Kit (Promega, USA) according to the manufacturer's recommendations.

### Statistical analysis

SPSS v.24.0 software (IBM, Armonk, NY, USA) was used for statistical analysis. The mean ± standard deviation was used to present the experimental results. Student’s t or one-way ANOVA was used to detect differences between groups, and the chi-square test was used to analyze the expression and clinical characteristics of LINC00571. *p* value < 0.05 was considered statistically significant.

## Results

### LINC00571 is upregulated in TNBC tissues and breast cancer cell lines

**Fig. 1:**
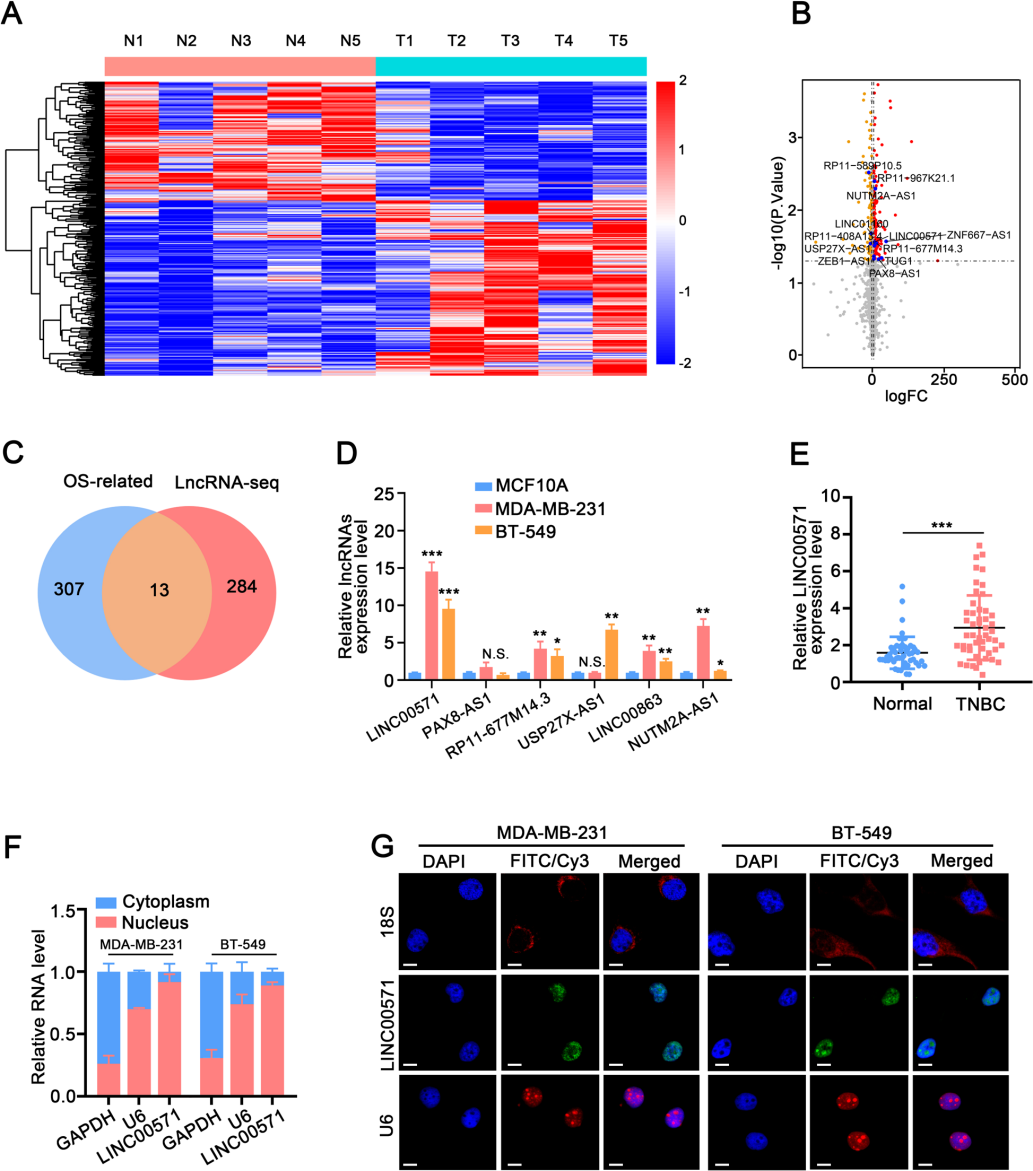
LINC00571 is upregulated in TNBC tissues and breast cancer cell lines. **A** Heatmap representing the diverse expression patterns of long non-coding RNAs across five pairs of triple-negative breast cancer tissues. **B** Volcano plot representing the diverse expression patterns of 13 long non-coding RNAs across five pairs of triple-negative breast cancer tissues. **C** Venn diagram illustrating the intersection between RNA-seq data derived from 5 pairs of triple-negative breast cancer tissues and prognostic data extracted from the TCGA database for triple-negative breast cancer. **D** PCR analysis revealing the relative abundance of lncRNAs in human breast cancer epithelial cell lines (MCF10A) and triple-negative breast cancer (TNBC) cell lines (MDA-MB-231, BT-549). GAPDH was utilized as an internal control. **E** Expression level assessment of LINC00571 in triple-negative breast cancer (TNBC) compared to adjacent normal breast tissues, performed using qRT-PCR analysis. **F** Determination of LINC00571 nuclear and cytoplasmic distribution by qRT-PCR analysis in MDA-MB-231 and BT-549 cells, Cytoplasmic and nuclear controls were GAPDH and U6, respectively. **G** RNA-FISH assay revealing the cytoplasmic localization of LINC00571 within MDA-MB-231 and BT-549 cells. Positive controls for cytoplasm (18S) and nucleus (U6) were labeled with Cy3 (red), while the LINC00571 probe was labeled with FITC (green). Nuclei were counterstained with DAPI (blue). scale bar :10μm. Statistical analyses are depicted in bar graphs. Data are presented as mean ± SD from three independent experiments. Significance levels are denoted as * for *p*<0.05, ** for *p*<0.01, and *** for *p*<0.001, as determined by the t-test

To identify significant lncRNAs that potentially participate in TNBC progression, we performed RNA-seq analysis to evaluate the lncRNA expression profiles using five pairs of TNBC clinical samples by. A heat map (Fig. 1A) and volcano plot (Fig. 1B) were generated to analyze the lncRNA expression profiles of TNBC versus paracancerous tissues (|logFC| >5, *p*< 0.05). The lncRNA-expression signatures from the TNBC samples in the Cancer Genome Atlas (TCGA) database were classified based on their correlation with the overall survival (OS) rate. We subsequently selected upregulated lncRNAs for intersection with OS via Venn diagram and identified 13 candidate lncRNAs (Fig. 1C). Prognosis analysis revealed that the expression levels of 6 lncRNAs (PAX8-AS1, LINC00571, RP11-677M14.3, USP27X-AS1:3, LINC00863, and NUTM2A-AS1) were associated with poor prognosis (Additional file 1). The expression levels of the 6 lncRNAs in TNBC cells were measured and LINC00571 was the most significantly upregulated lncRNA. Thus, LINC00571 was further explored as a target molecule (Fig. 1D).

LINC00571 is located on human chromosome 13 and consists of 6 exons with a total length of 521nt (Additional file 2A). The full-length sequence, its secondary structure based on minimum free energy (MFE) and the analysis of its potential coding ability are shown in Additional file 2B-E, respectively. Consistent with the RNA-seq results, LINC00571 was significantly upregulated in TNBC tissues (Fig. 1E). To determine the subcellular distribution of LINC00571, we performed a nuclear/cytoplasmic RNA fractionation assay. LINC00571 was mainly located in the nucleus in both TNBC and non-TNBC cells (Fig. 1F and Additional file 2F). These findings were confirmed by fluorescence in situ hybridization (FISH) analysis (Fig. 1G and Additional file 2G). To determine understand the relationship between the expression level of LINC00571 and the clinicopathological characteristics of TNBC patients, we extracted the clinical and pathological information of breast cancer patients and summarized the findings in Table 1. A positive correlation was revealed between the elevated expression of LINC00571 and tumor differentiation, lymphatic metastasis, distant metastasis, and Ki-67 levels in TNBC patients. In summary, LINC00571 is significantly upregulated in TNBC and is linked to an unfavorable prognosis in TNBC patients.

**Table 1:** Clinicopathological correlations of LINC00571 expression in triple-negative breast cancer

	**Expression of LINC00571**			***p*-value**
	**Low**	**High**	**Total**	
Age(y)				
≤ 60	22	26	42	0.456
> 60	20	32	58	
Tumor differentiation				
Well	22	10	32	<0.001
Moderate	14	24	38	
Poor	6	24	30	
Lymphatic metastasis				
Positive	10	36	46	<0.001
Negative	32	22	54	
Distant metastasis				
Positive	10	30	40	0.005
Negative	32	28	60	
Ki-67				
Low	28	22	50	0.005
High	14	36	50	

### LINC00571 regulates the proliferation and apoptosis of TNBC cells

**Fig. 2:**
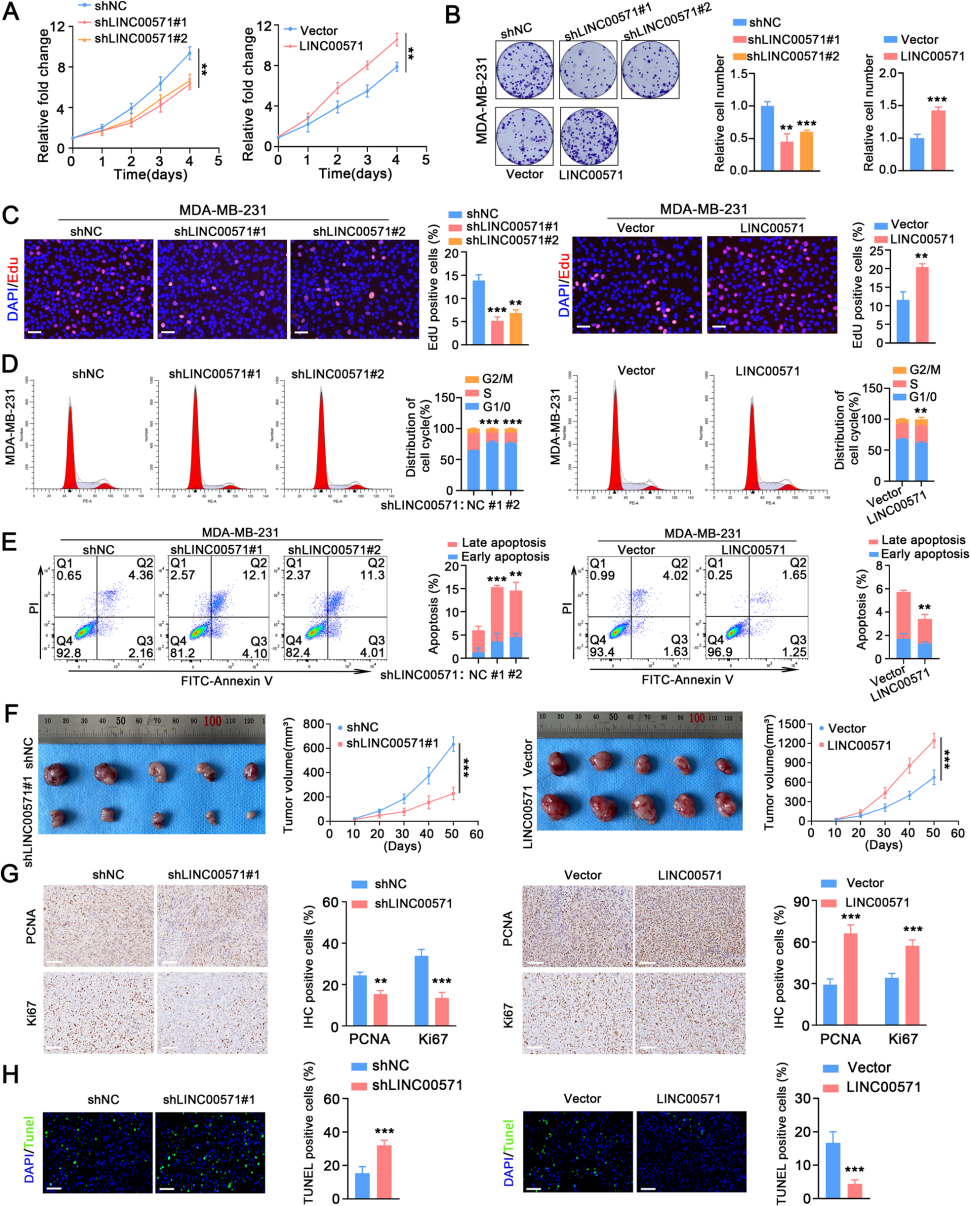
LINC00571 regulates proliferation and apoptosis of TNBC cells. **A**-**C** Cellular proliferation rates were assessed using CCK-8, colony formation, and EdU assays, revealing the impact of shNC or shLINC00571 and vector or LINC00571 in MDA-MB-231 cells, *n*=3. scale bar: 50μm. **D** Flow cytometry-based cell cycle analysis conducted on MDA-MB-231 cells stained with propidium iodide (PI), *n*=3. **E** Apoptosis evaluation carried out through a flow cytometry assay on MDA-MB-231 cells stained with Annexin V (FITC) and propidium iodide (PI), *n*=3. **F** Photographs of xenograft s captured with a digital camera. MDA-MB-231 cells were subcutaneously injected into BALB/c athymic nude mice, *n*=5. Volume was monitored every ten days and calculated as volume = length × (width)^2^/2. **G** Immunohistochemical images showing Ki67 and PCNA staining in shNC or shLINC00571 groups (left) and vector or LINC00571 groups (right), scale bar: 100μm. Quantification of positive Ki67 and PCNA, *n* = 5. **H** Immunofluorescence images illustrating TUNEL staining in shNC or shLINC00571 groups (left) and vector or LINC00571 groups (right), scale bar: 60μm. Quantification of positive TUNEL, *n* = 5. Statistical analyses are depicted in bar graphs. Data are presented as mean ± SD. Significance levels are denoted as * for *p*<0.05, ** for *p*<0.01, and *** for *p*<0.001, as determined by the t-test

To determine the biological function of lncRNAs in breast cancer cells, we first developed stable LINC00571 knockdown and overexpression models in MDA-MB-231 and BT-549 cells (Additional file 3A, B). The CCK8, colony formation and EdU assays results indicated that knockdown of LINC00571 significantly reduced the proliferation of MDA-MB-231 and BT-549 cells compared to the control group, while overexpression of LINC00571 significantly increased cell proliferation (Fig. 2A-C and Additional file 3C-E). Moreover, cell cycle analysis was performed by flow cytometry, the results of which revealed that LINC00571 knockdown led to a lower percentage of TNBC cells in S phase and a greater proportion of TNBC cells in G1 phase compared with the control group. These findings suggest that LINC00571 knockdown caused G1 arrest in TNBC cells. In contrast, LINC00571 overexpression significantly promoted G1-S phase cell cycle transition (Fig. 2D and Additional file 3F). Flow cytometry analysis showed that the apoptosis rate of MDA-MB-231 and BT-549 cells with LINC00571 knockdown was significantly greater than that of the control cells, while that in LINC00571 overexpressing cells was significantly decreased (Fig. 2E and Additional file 3G). These results indicated that LINC00571 regulated the proliferation and apoptosis of TNBC cells.

In addition, in vivo xenograft tumor experiments revealed that the volume of xenograft tumors generated from LINC00571 knockdown TNBC cells were significantly lower than those generated from control cells, whereas LINC00571 overexpression increased the volume of xenograft tumors (Fig. 2F). Immunohistochemical (IHC) analysis revealed that LINC00571 knockdown reduced the expression of Ki67 and PCNA in xenograft tumors, whereas LINC00571 overexpression increased the expression of these genes (Fig. 2G). TUNEL assay results showed that LINC00571 knockdown reduced apoptosis in xenograft tumors, while LINC00571 overexpression increased apoptosis (Fig. 2H). These results suggest that LINC00571 may promote TNBC progression.

### LINC00571 promotes the progression of TNBC through the TCA signaling pathway

**Fig. 3:**
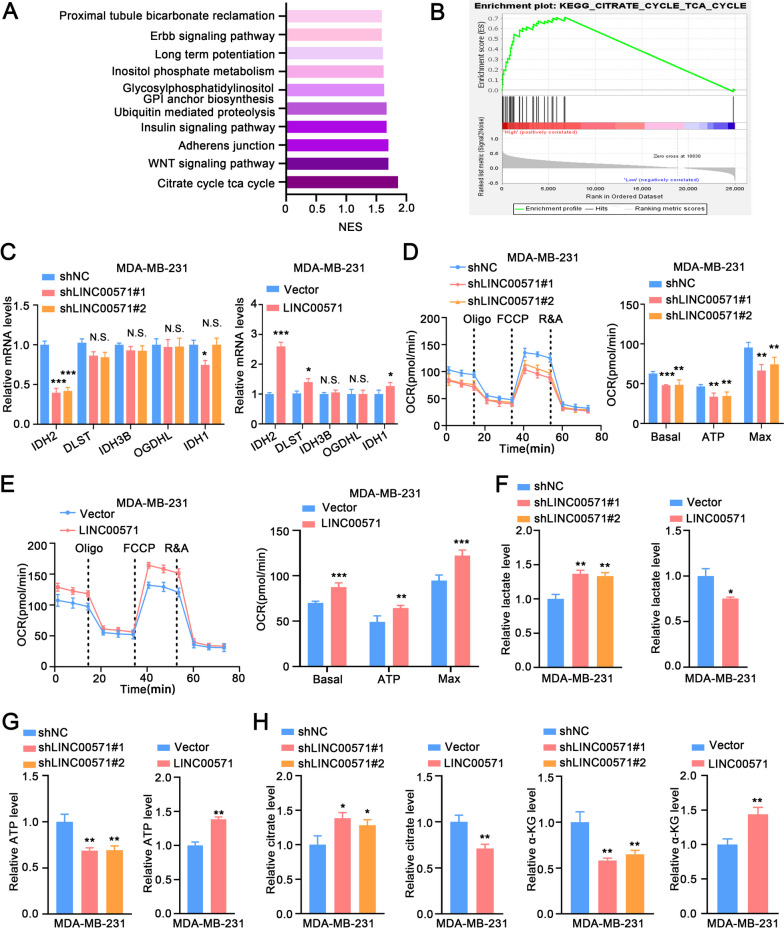
LINC00571 promotes the progression of TNBC by TCA signaling pathway. **A** Gene set enrichment analysis (GSEA) of the TCGA dataset using the TCGA dataset revealed the top 10 pathways associated with high expression of LINC00571. **B** GSEA plots showed a positive correlation between LINC00571 expression and the set of upregulated genes in tricarboxylic acid (TCA) cycle within triple-negative breast cancer. **C** PCR analysis revealed the expression profile of corresponding genes in MDA-MB-231 cells with LINC00571 knockdown (left) or LINC00571 overexpression (right), *n* = 3. **D** Left, oxygen consumption rate (OCR) was analyzed in MDA-MB-231 cells with LINC00571 knockdown (*n* = 4). Right, basal respiration, ATP-coupled respiration and maximal respiration (*n*= 4). **E** Left, oxygen consumption rate (OCR) was analyzed in MDA-MB-231 cells with LINC00571 overexpression (*n* = 4). **F**-**G** Relative lactate level (**F**) and relative ATP level (**G**) in MDA-MB-231 cells with LINC00571 knockdown (left) or LINC00571 overexpression (right) were shown, *n* = 3. (H) Relative lactate level (**F**) and relative ATP level (**G**) in MDA-MB-231 cells with LINC00571 knockdown (left) or LINC00571 overexpression (right) were shown, *n* = 3. Statistical analyses are depicted in bar graphs. Data are presented as mean ± SD. Significance levels are denoted as * for *p*<0.05, ** for *p*<0.01, and *** for *p*<0.001, as determined by the t-test

Based on information from the TCGA database, we classified high and low LINC00571 expression groups and performed Gene Set Enrichment Analysis (GSEA) to explore the regulatory pathways of LINC00571. GSEA analysis indicated that the increased expression of LINC00571 activated the TCA cycle-associated pathway in TNBC (Fig. 3A, B). Consequently, further analysis of the LINC00571 high-expression group and LINC00571 low-expression group to generate a heatmap of TCA cycle pathway gene expression (Additional file 4A). IDH2, DLST, IDH3B, OGDHL, and IDH1 were the top 5 candidate genes considered for investigation. We subsequently identified that IDH2 as a target molecule by detecting the mRNA levels of the above 5 candidate genes after up- and down-regulation of LINC00571 (Fig. 3C and Additional file 4B). Knockdown of LINC00571 decreased the oxygen consumption rate (OCR) in TNBC cells, while overexpression of LINC00571 increased the OCR (Fig. 3D, E and Additional file 4C, D). LINC00571 knockdown significantly elevated lactate levels, and disrupted ATP generation, whereas LINC00571 overexpression had the opposite effect (Fig. 3F and G, Additional file 4E, F). We then measured TCA-related metabolites, and observed an increase in citrate levels along with a reduction in α-KG in TNBC cells with LINC00571 knockdown, overexpression of LINC00571 resulted in the opposite trend (Fig. 3H and Additional file 4G). In summary, these data demonstrated a role for LINC00571 in regulating both TCA cycle enzyme expression and cellular bioenergetics in TNBC cells.

### LINC00571 interacts with the HNRNPK and ILF2 proteins

**Fig. 4:**
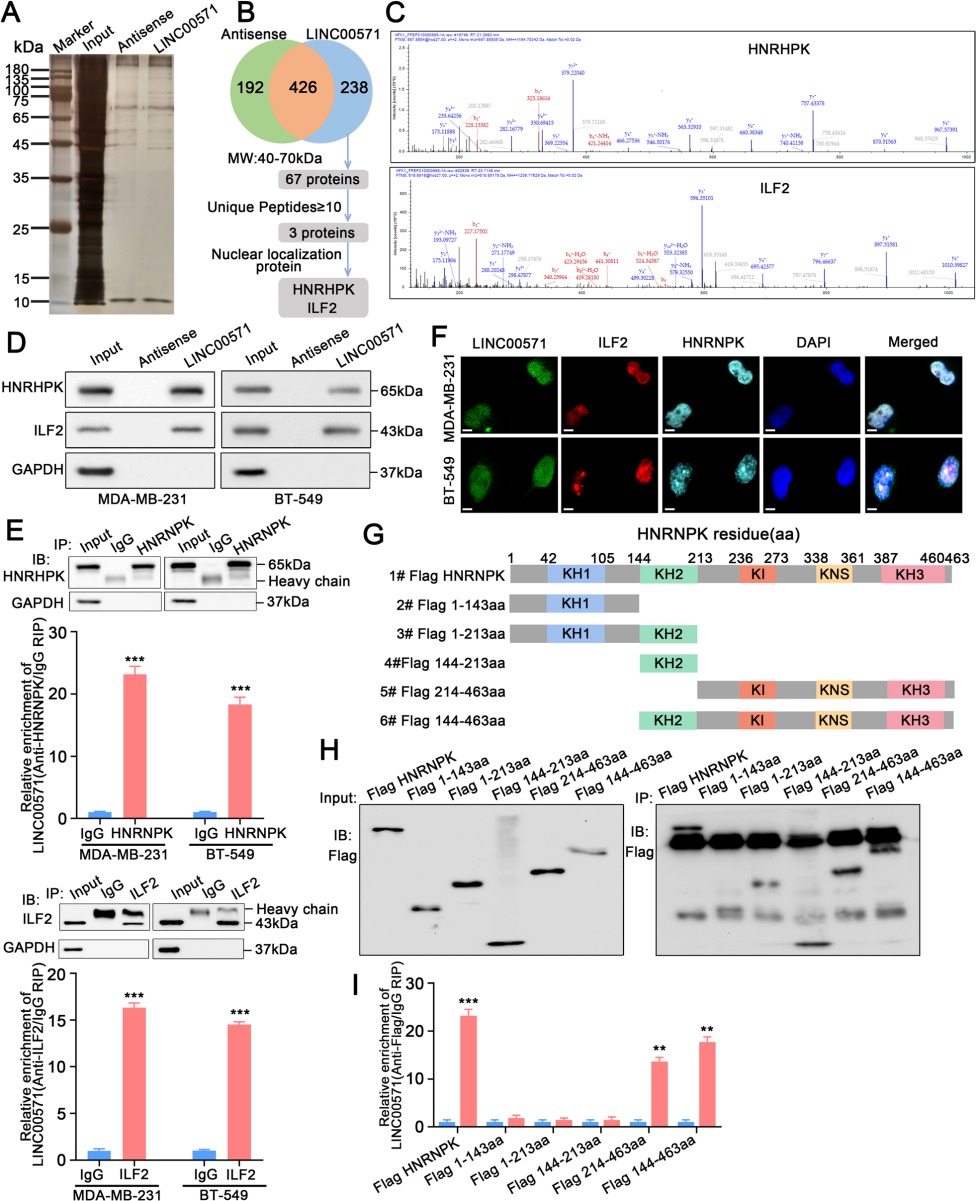
LINC00571 interacts with HNRNPK protein and ILF2 protein. **A** Silver staining unveiled proteins interacting with LINC00571, with biotin-labeled sense or antisense LINC00571 probes used for RNA-protein pull-down against MDA-MB-231 cell lysates. **B** A simplified flowchart outlined the systematic screening process used to identify proteins that interacted with LINC00571. **C** Mass spectrometry analysis revealed HNRNPK peptides and ILF2 peptides pulled down by LINC00571 sense probes. **D** Immunoblot analyses were performed for HNRNPK and ILF2 on biotin-labeled sense and antisense LINC00571 probe pull-down eluates from MDA-MB-231 and BT-549 cell lysates, with GAPDH as a loading control. **E** RNA immunoprecipitation (RIP) was conducted on MDA-MB-231 and BT-549 cells using HNRNPK and IgG antibody or ILF2 and IgG antibody. The precipitates underwent immunoblot analysis with HNRNPK and GAPDH antibody or ILF2 and GAPDH antibody. HNRNPK or ILF2 enrichment of LINC00571 relative to IgG enrichment values was quantified by qRT-PCR. **F** RNA-FISH and immunofluorescence staining assays revealed subcellular co-localization of LINC00571 (green), ILF2 (red), and HNRNPK (cyan), along with nuclear staining using DAPI (blue). scale bar: 10μm (**G**) Schematic representation of HNRNPK with functional protein domains. HNRNPK had been truncated within regions: 1-143aa, 1-213aa, 144-213aa, 214-463aa, and 144-463aa. **H**-**I** Relative enrichment of endogenous LINC00571 in truncated HNRNPK RIP was measured by qRT-PCR, following MDA-MB-231 cells transfected with 3xFlag-HNRNPK truncations. Statistical analyses are depicted in bar graphs. Data are presented as mean ± SD from three independent experiments. Significance levels are denoted as * for *p*<0.05, ** for *p*<0.01, and *** for *p*<0.001, as determined by the t-test

The published literature shows that lncRNAs participate in cellular processes by interacting with RBPs [16–19]. Therefore, to explore the RBPs that interact with LINC00571, we used biotin- LINC00571 to analyze the cellular extracts of MDA-MB-231 cells via an RNA pull-down assay. The captured proteins were analyzed by silver staining (Fig. 4A). Figure 4B shows the screening process for proteins bound to LINC00571. The major differential bands that precipitated from the MDA-MB-231 cell lysates were identified as HNRNPK and ILF2 through mass spectrometry (Fig. 4C). The interactions between LINC00571 and HNRNPK, and between LINC00571 and ILF2 were further validated through RNA pull-down (Fig. 4D and Additional file 5A) and RIP analysis (Fig. 4E). In addition, we confirmed the colocalization of endogenously expressed LINC00571, HNRNPK, and ILF2 in the nucleus by immunofluorescence in situ hybridization analysis (Fig. 4F). HNRNPK contains three K-homology (KH) domains that mediate nucleic acid binding. We generated a truncated HNRNPK protein based on the location of the KH region (Fig. 4G). Analysis of the protein domain localization revealed that the KH3 domain of HNRNPK was essential for its binding to LINC00571. Deletion of the 214-463 aa region of HNRNPK significantly reduced its binding rate with LINC00571 (Fig. 4H, I). In summary, these data confirmed that LINC00571 interacts with HNRNPK through the KH3 domain of HNRNPK.

### HNRNPK binds with ILF2 to promote the stabilization of ILF2

**Fig. 5:**
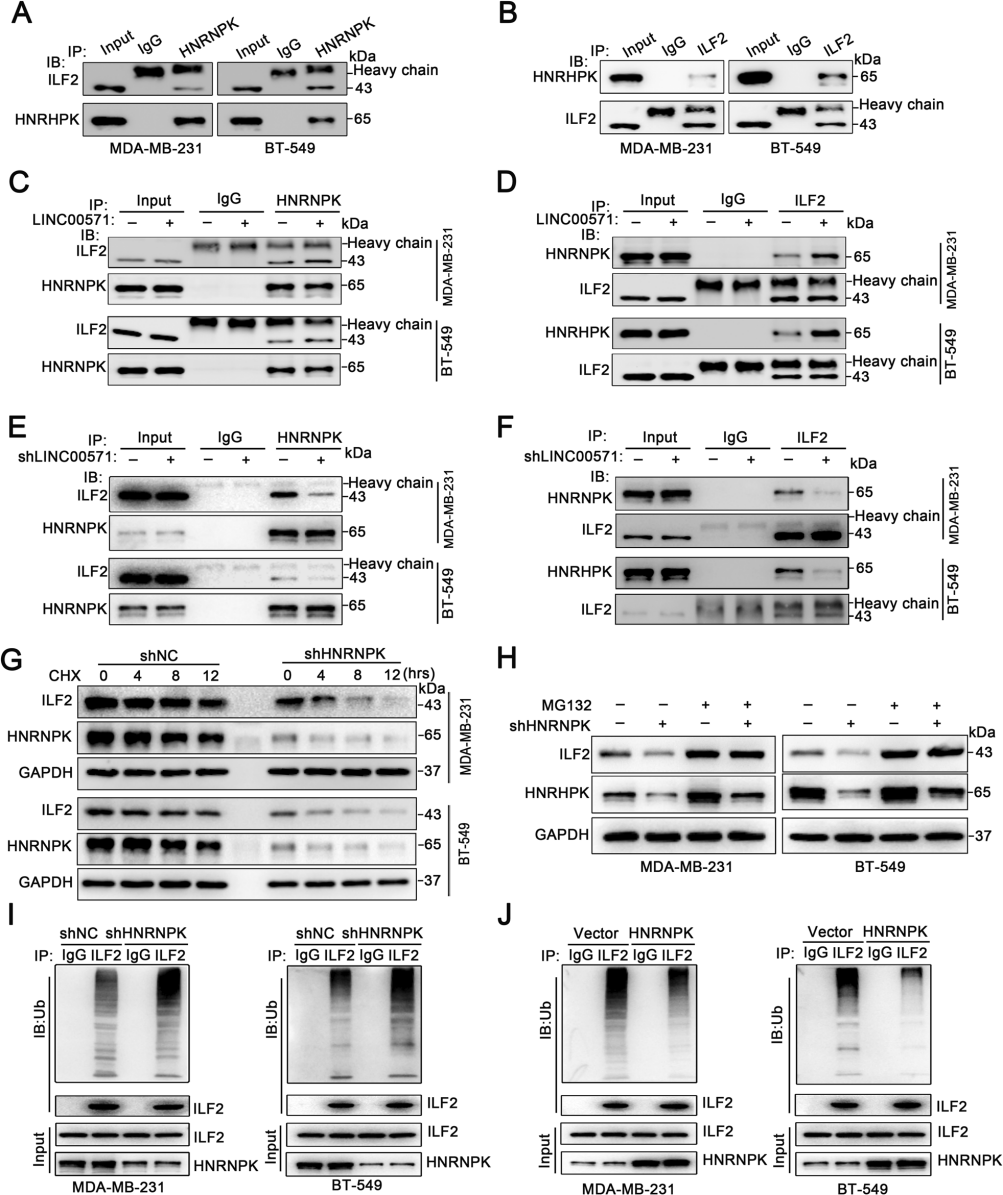
HNRNPK binds with ILF2 to promote the stabilization of ILF2. **A**-**B** Coimmunoprecipitation (Co-IP) assays were conducted using anti-HNRNPK (**A**) or anti-ILF2 (**B**) antibodies in TNBC cells, followed by immunoblot (IB) analysis for HNRNPK and ILF2. Immunoglobulin G (IgG) was utilized as a negative control antibody for immunoprecipitations. **C**-**F** Co-IP and IB assays demonstrated that LINC00571 overexpression led to an increase in the binding of HNRNPK and ILF2 (C-D), while LINC00571 knockdown resulted in decreased HNRNPK and ILF2 binding (E-F). **G** Immunoblot analysis depicted the cycloheximide (CHX) chase analysis of ILF2 protein degradation at indicated time points (t=0, 4, 8, 12h) in TNBC cells with or without HNRNPK. **H** Immunoblot analysis revealed the levels of ILF2 and HNRNPK in HNRNPK knockdown cells treated with vehicle control or MG132 (10 μM) for 12 hours in MDA-MB-231 cells (left) and BT-549 cells (right). **I** IP and IB demonstrated that knockdown of HNRNPK inhibited the ubiquitination of ILF2 in MDA-MB-231 cells (left) and BT-549 cells (right) treated with MG132. **J** IP and IB assays illustrated that overexpression of HNRNPK promoted the ubiquitination of ILF2 in MDA-MB-231 cells (left) and BT-549 cells (right) treated with MG132

Based on the above results, LINC00571, HNRNPK and ILF2 constitute a complex. To further understand the role of the two RBPs in the complex, we performed Co-IP experiments and verified that HNRNPK binds to ILF2 (Fig. 5A, B), and that the binding rate increased significantly after overexpression of LINC00571 (Fig. 5C, D), and decreased significantly after knockdown of LINC00571 (Fig. 5E, F). In addition, we found that knockdown or overexpression of LINC00571 had no effect on the expression levels of the RBPs (HNRNPK and ILF2) at the mRNA and protein levels (Additional file 5B-E). Therefore, we speculate that LINC00571 acts as a scaffold for binding HNRNPK and ILF2. Furthermore, ILF2 was rapidly degraded in the HNRNPK-knockdown group (Fig. 5G and Additional file 5F). To elucidate the mechanism of ILF2 degradation, we treated TNBC cells with the proteasome inhibitor MG132. The results suggested that treatment with MG132 increased the protein level of ILF2. Notably, MG132 treatment greatly rescued the decrease in the ILF2 protein level in HNRNPK-knockdown TNBC cells (Fig. 5H), suggesting that a decrease in the protein stability of ILF2, which is regulated through the ubiquitin–proteasome pathway, was induced by HNRNPK downregulation. The ubiquitination of ILF2 was markedly elevated in TNBC cells with silenced HNRNPK (Fig. 5I). The overexpression of HNRNPK led to a reduction in the ubiquitination of ILF2 (Fig. 5J and Additional file 5G). These data suggest that HNRNPK promotes the stability of the ILF2 protein.

### ILF2 promotes TNBC progression by regulating IDH2 transcription

**Fig. 6:**
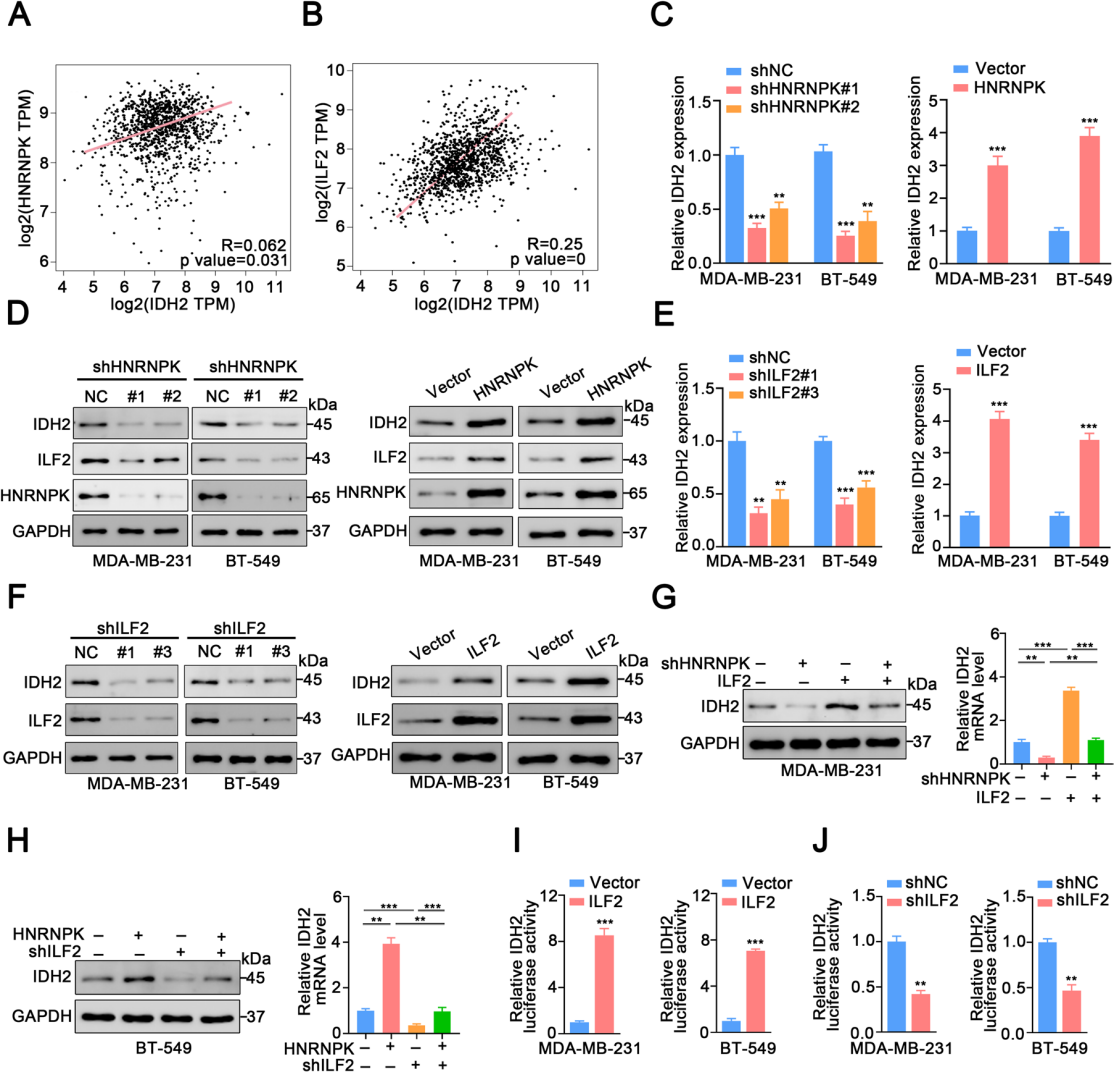
ILF2 promoted TNBC progression by regulating the transcription of IDH2. **A** Correlation analysis between IDH2 and HNRNPK expression in BRCA s and normals using TCGA dataset at GEPIA. **B** Correlation analysis between IDH2 and ILF2 expression in BRCA tumors and normals using TCGA dataset at GEPIA. **C** qRT-PCR assay revealed the expression level of IDH2 in TNBC cells with HNRNPK knockdown (left) or HNRNPK overexpression (right). **D** Immunoblot analysis displayed the levels of IDH2, HNRNPK, and ILF2 in TNBC cells with HNRNPK knockdown (left) or HNRNPK overexpression (right), using GAPDH as a loading control. **E** qRT-PCR assay showcased the expression level of IDH2 in TNBC cells with ILF2 knockdown (left) or ILF2 overexpression (right). **F** Immunoblot analysis depicted the levels of IDH2 and ILF2 in TNBC cells with ILF2 knockdown (left) or ILF2 overexpression (right), with GAPDH as a loading control. **G** Immunoblot analysis displayed the levels of IDH2 in MDA-MB-231 cells co-transfected with shHNRNPK and ILF2 (left). qRT-PCR assay illustrated the expression level of IDH2 in MDA-MB-231 cells co-transfected with shHNRNPK and ILF2 (right). **H** Immunoblot analysis showed the levels of IDH2 in BT-549 cells co-transfected with HNRNPK and shILF2 (left). qRT-PCR assay revealed the expression level of IDH2 in BT-549 cells co-transfected with HNRNPK and shILF2 (right). **I**-**J** Impact of ILF2 overexpression (**I**) or knockdown (**J**) on luciferase activity in the IDH2 promoter region. Statistical analyses are depicted in bar graphs. Data are presented as mean ± SD from three independent experiments. Significance levels are denoted as * for *p*<0.05, ** for *p*<0.01, and *** for *p*<0.001, as determined by the t-test

Considering that LINC00571 promotes the progression of TNBC by mediating IDH2 in the TCA signaling pathway, we explored the relationships between HNTNPK and IDH2, and between ILF2 and IDH2. Initially, we conducted an in-depth analysis of the clinical characteristics associated with IDH2 status by leveraging publicly available data. GEPIA database analysis revealed that IDH2 expression was greater in breast cancer than in normal tissue (Additional file 6A). Subsequently, we found that HER2 positive, triple negative patients and luminal type patients had higher IDH2 expression than normal patients (Additional file 6B). The expression level of IDH2 was positively correlated with tumor stage and lymph node stage (Additional file 6C, D). In addition, breast cancer patients with high IDH2 levels had a worse prognosis in overall survival (OS) (Additional file 6E), recurrence free survival (RFS) (Additional file 6F), and distant metastasis-free survival (DMFS) (Additional file 6G). Furthermore, Spearman correlation analysis demonstrated that HNRNPK and ILF2 expression was positively correlated with IDH2 expression (Fig. 6A, B). The results showed that the expression level of IDH2 was significantly downregulated after knockdown of HNRNPK (Fig. 6C, D). In contrast, the expression level of IDH2 was significantly upregulated after overexpression of HNRNPK (Fig. 6C and D). Similarly, knockdown of ILF2 resulted in reduced IDH2 expression, whereas overexpression of ILF2 led to increased IDH2 expression. (Fig. 6E, F and Additional file 5H, I).

We observed a downregulation of IDH2 mRNA and protein expression in TNBC cells with HNRNPK knockdown. but these effects were reversed in the TNBC cells co-transfected with HNRNPK knockdown and IFL2 overexpression (Fig. 6G). The opposite trend in IDH2 expression was also observed in TNBC cells with simultaneous HNRNPK overexpression and ILF2 knockdown to TNBC cells (Fig. 6H). Studies have shown that ILF2 promotes gene transcription, and we hypothesized that it could bind to the IDH2 gene upstream to regulate its transcription. As expected, the relative firefly luciferase activity of IDH2 was significantly enhanced in TNBC cells overexpressing ILF2 (Fig. 6I). Moreover, the relative firefly luciferase activity of IDH2 was obviously reduced in TNBC cells after ILF2 elimination (Fig. 6J). Collectively, these findings provide evidence that ILF2 facilitates the progression of TNBC by modulating the transcription of IDH2.

### ILF2 promotes TNBC progression via enhanced TCA flux

**Fig. 7:**
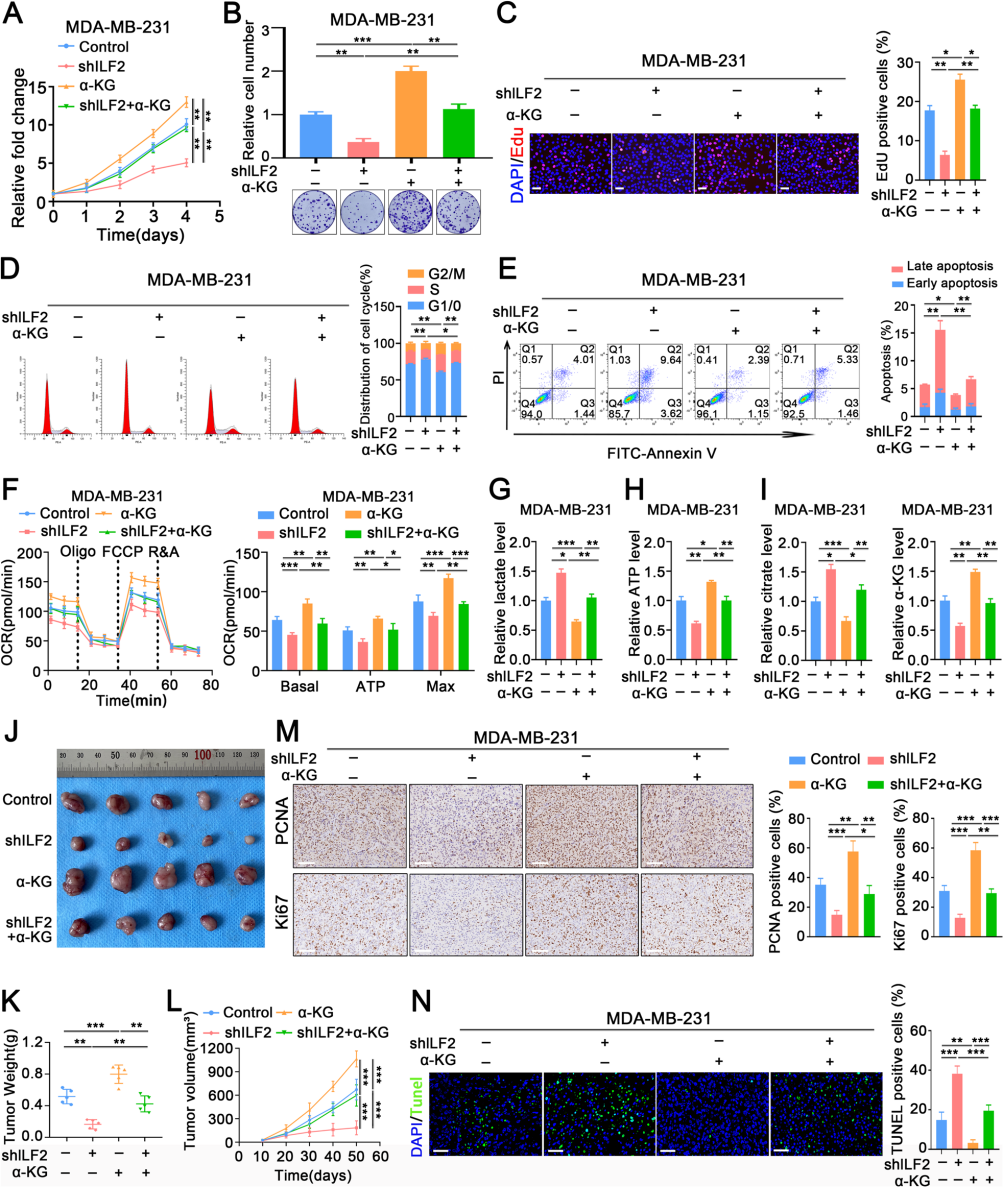
The addition of α-KG rescues the phenotypes induced by ILF2 knockdown. (**A**-**C**) Proliferation rate status of MDA-MB-231 cells was assessed through CCK-8, colony formation assays, and EdU assays, *n* = 3. scale bar: 50μm. **D** Cell cycle analysis was conducted via flow cytometry, involving propidium iodide (PI) staining on MDA-MB-231 cells, *n* = 3. **E** Apoptosis detection was performed using a flow cytometry assay. Annexin V and propidium iodide (PI) staining were employed on MDA-MB-231 cells, *n* = 3. **F** Left, oxygen consumption rate (OCR) on addition of oligomycin (Oligo), fluorocarbonyl cyanide phenylhydrazone (FCCP) and rotenone plus antimycin A (R&A) (*n* = 4). Right, basal respiration, ATP-coupled respiration and maximal respiration (*n*= 4). **G**-**H** Relative lactate level (**G**) and relative ATP level (**H**) in MDA-MB-231 cells with ILF2 knockdown and α-KG supplementation were shown, *n* = 3. (**I**) Relative lactate level (left) and relative ATP level (right) in MDA-MB-231 cells with ILF2 knockdown and α-KG supplementation were shown, *n* = 3. **J**-**N** In vivo studies involved BALB/c athymic nude mice subcutaneously injected with MDA-MB-231 cells, *n* = 5. Images of xenograft s were captured using a digital camera (**J**). Tumor weight was measured on day 50 (**K**). Tumor volume was measured at ten-day intervals, calculated as volume = length × (width)^2^/2 (**L**). Immunohistochemical images showing Ki67 and PCNA staining in control, shILF2-, α-KG-, and shILF2 combined plus α-KG-treated groups. scale bar: 100μm (**M**). Immunofluorescence images showcased TUNEL staining in control, shILF2-, α-KG-, and shILF2 combined plus α-KG-treated groups. scale bar: 60μm (**N**). Statistical analyses are depicted in bar graphs. Data are presented as mean ± SD. Significance levels are denoted as * for *p*<0.05, ** for *p*<0.01, and *** for *p*<0.001, as determined by the t-test

IDH2 is an NADP^+/-^ dependent mitochondrial enzyme that catalyzes the interconversion between isocitrate and α-KG in the TCA cycle, and thus plays a major role in cellular metabolism [34, 35]. We further verified the role of the ILF2/IDH2 axis in TNBC progression. α-KG was added to the medium of shILF2-treated MDA-MB-231 and BT-549 cells. Subsequently, CCK-8, colony formation, cell cycle, EdU, and apoptosis assays were performed on control, shILF2-, α-KG-, and shILF2 combined plus α-KG-treated MDA-MB-231 cells. The results indicated that the addition of α-KG rescued the inhibitory effect of ILF2 knockdown on cell viability (Fig. 7A-E). Notably, compared with that in the control group, the cellular OCR was decreased, the lactate level of lactate was elevated, and disrupt ATP generation was disrupted in ILF2-knockdown MDA-MB-231 cells, these effects were rescued by the addition of α-KG (Fig. 7F-I). Moreover, the addition of α-KG reversed the inhibitory effect of ILF2 knockdown on tumor size and growth (Fig. 7J-L). Additionally, IHC and TUNEL assays also showed that ILF2 knockdown markedly decreased proliferation and promoted apoptosis, but these effects were reversed in MDA-MB-231 cells with ILF2 knockdown and α-KG addition (Fig. 7M, N). IDH2 knockdown inhibited the enhanced proliferation and increased apoptosis caused by ILF2 overexpression (Additional file 7A-E). Moreover, compared with that in the control group, the cellular OCR was increased, the lactate level was reduced, and ATP generation was enhanced in ILF2-overexpressing TNBC cells, these effects were reversed by knocking down IDH2 (Additional file 7F-J). The present results demonstrated that ILF2 promoted TNBC progression by enhancing TCA flux.

### The LINC00571/HNRNPK/ILF2-TCA cycle axis promotes TNBC progression

**Fig. 8.**
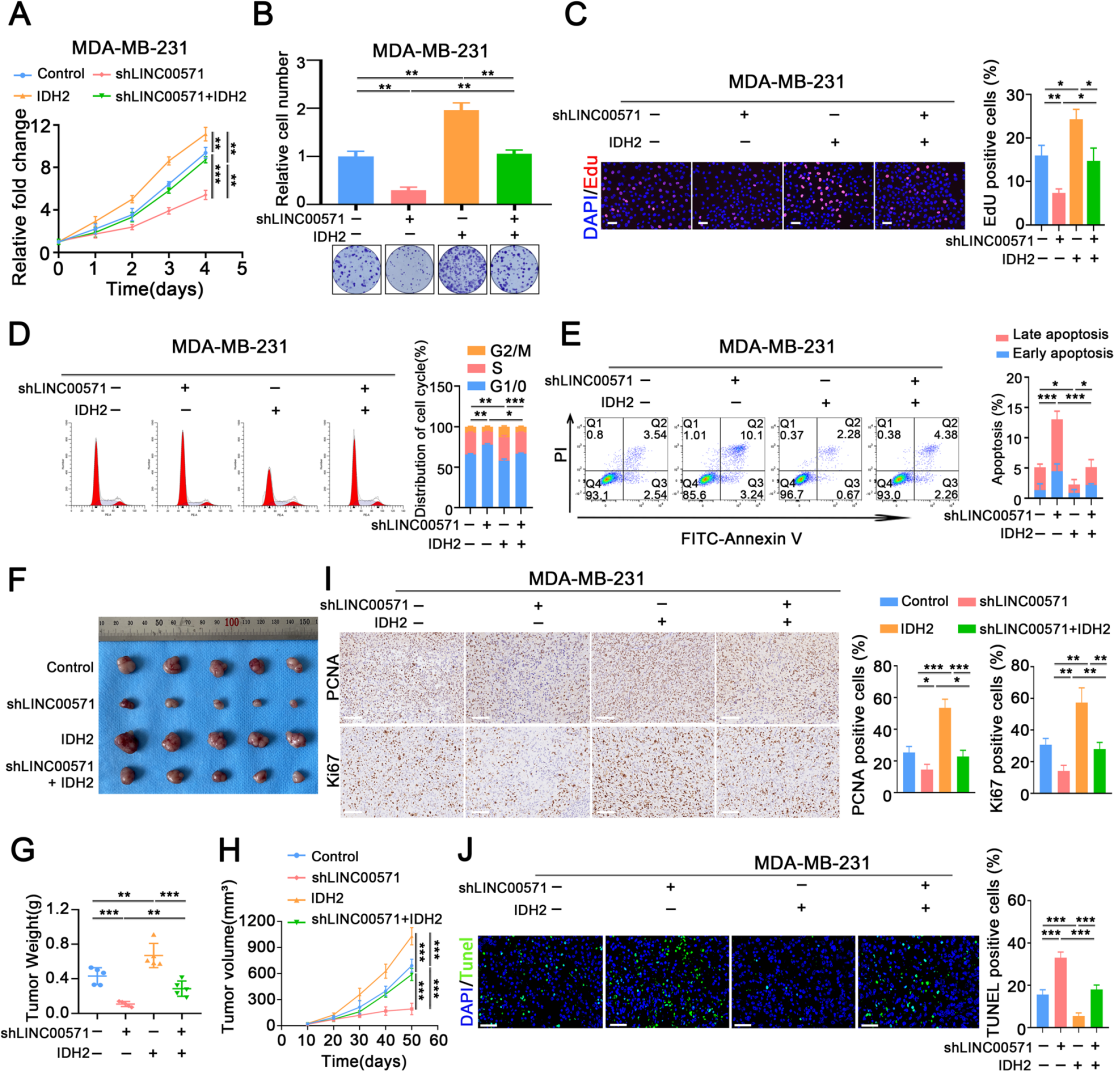
LINC00571 mediates IDH2 expression to regulate the progression of TNBC cells. **A**-**C** Proliferation status of MDA-MB-231 cells co-transfected with shLINC00571 and IDH2 was assessed using CCK-8, colony formation assays, and EdU assays, *n* = 3. scale bar: 50μm. (**D**) Cell cycle analysis was conducted via flow cytometry, and MDA-MB-231 cells were stained with propidium iodide (PI), *n* = 3. **E** Apoptosis was detected using a flow cytometry assay, with Annexin V and propidium iodide (PI) staining in MDA-MB-231 cells, *n* = 3. **F**-**J** In vivo studies involved subcutaneous injection of MDA-MB-231 cells co-transfected with shLINC00571 and IDH2 into BALB/c athymic nude mice, *n* =5. Images of xenograft tumors were captured using a digital camera (**F**). Tumor weight was measured on day 50 (**G**). Tumor volume was monitored every ten days, calculated using the formula: volume = length × (width)^2^/2 (**H**). Immunohistochemical images of Ki67 and PCNA staining were conducted for control, shLINC00571-, IDH2-, and shLINC00571 combined plus IDH2-treated groups. scale bar: 100μm (**I**). Immunofluorescence images illustrated TUNEL staining in control, shLINC00571-, IDH2-, and shLINC00571 combined plus IDH2-treated groups. scale bar: 60μm (**J**). Statistical analyses are depicted in bar graphs. Data are presented as mean ± SD from three independent experiments. Significance levels are denoted as * for *p*<0.05, ** for *p*<0.01, and *** for *p*<0.001, as determined by the t-test

To further explore whether LINC00571 plays a biological role via IDH2, a series of rescue experiments were carried out in TNBC cells co-transfected with LINC00571 knockdown and IDH2 overexpression plasmids. The results revealed that overexpression of IDH2 could markedly reverse the inhibitory effects of LINC00571 downregulation on the proliferation and cell cycle progression of MDA-MB-231 cells (Fig. 8A-D), and the promoting role of LINC00571 downregulation in the apoptosis of TNBC cells (Fig. 8E). LINC00571 upregulation promoted cell proliferation, cell cycle progression in BT-549 cells, and concurrently inhibiting apoptosis, which could be markedly reversed upon knockdown of IDH2 (Additional file 8A-E). In addition, the weight and volume of MDA-MB-231 subcutaneous and orthotopic breast tumors in BALB/c nude mice were significantly reduced by knocking down LINC00571. Overexpression of IDH2 reversed these effects (Fig. 8F-H and Additional file 9A-C). Additionally, IHC and TUNEL assays also showed that LINC00571 knockdown markedly decreased proliferation and promoted apoptosis, and these effects were reversed in MDA-MB-231 cells co-transfected with LINC00571 knockdown and IDH2 overexpression plasmids (Fig. 8I, J).

**Fig. 9:**
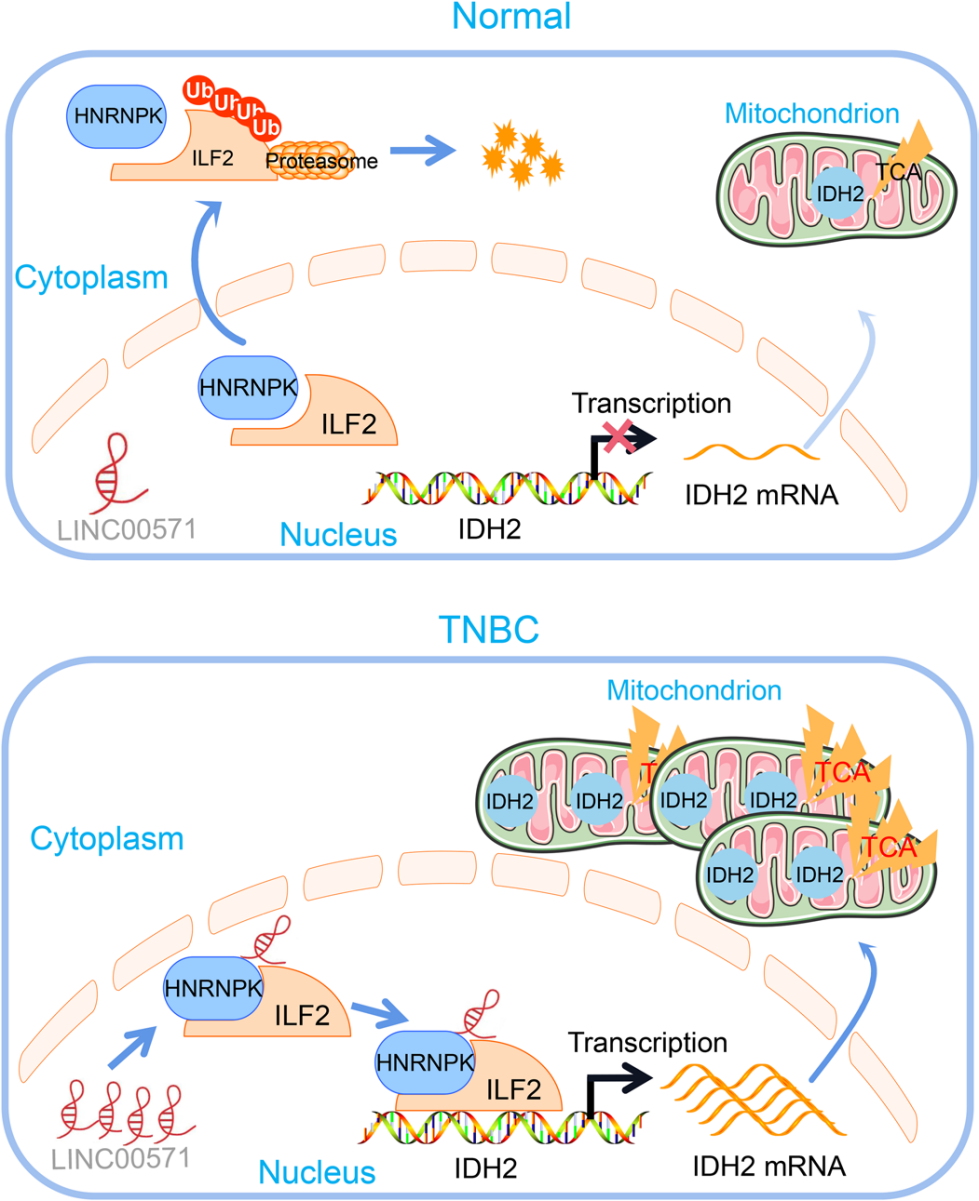
This illustration shows that the lncRNA LINC00571 upregulates the tricarboxylic acid cycle (TCA) in breast cancer, thereby promoting breast cancer progression through the dysregulation of IDH2 expression via the HNRNPK/IL2 axis

Supplementation with α-KG significantly mitigated the reduction in cell viability induced by LINC00571 deficiency. Downregulation of LINC00571 resulted in decreased proliferation, G1 phase cell cycle arrest, and increased apoptosis in MDA-MB-231 cells. These effects were reversed by α-KG supplementation (Additional file 10A-E). Moreover, LINC00571 knockdown significantly reduced the weight and volume of subcutaneous tumors in mice, and these effects were restored by α-KG supplementation (Additional file 10F-H). Additionally, LINC00571 knockdown markedly decreased proliferation and increased apoptosis, as observed through IHC and TUNEL assays, respectively. These effects were reversed by α-KG supplementation (Additional file 10I, J). In conclusion, our study demonstrated that LINC00571 was highly expressed in breast cancer, and promoted cancer cell proliferation through the HNRNPK/ILF2-TCA cycle axis (Fig. 9).

## Discussion

With the advances in deep sequencing technology, numerous lncRNAs have been identified in mammalian cells and tissues [36, 37]. However, the biological mechanisms underlying lncRNAs in TNBC have not been comprehensively elucidated. This study revealed that LINC00571 interacted with the proteins HNRNPK and ILF2. LINC00571 acts as a molecular scaffold that regulates ILF2 stability through HNRNPK. This interaction leads to enhanced IDH2 transcription, promoting the progression of TNBC. These findings unveil a novel regulatory role for LINC00571 in TNBC development, highlighting its significance in breast cancer progression.

Emerging research has consistently indicated the aberrant expression of lncRNAs in cancer [38–40], highlighting their potential as valuable targets for cancer diagnosis and treatment. The functionality of lncRNAs is closely tied to their cellular localization, relative abundance, and interactions with other molecules [41–43]. In this study, we elucidated the predominant nuclear localization of LINC00571. Gain- and loss-of-function studies demonstrated that LINC00571 promotes TNBC cell viability. LINC00571, a novel long non-coding RNA, functions as a scaffold molecule that forms a complex with HNRNPK and ILF2, thereby providing a novel model for understanding the regulatory mechanisms of TNBC. We also observed a minor fraction of LINC00571 in the cytoplasm, the function of which requires further exploration.

HNRNPK, which features the KH 1, KH 2, and KH 3 structural domains, plays a critical role in nucleic acid recognition, promoting its binding to both RNA and DNA [44]. Recent research revealed that the long non-coding RNA SINEUP facilitates the assembly of translation initiation complexes via interactions with PTBP1 and HNRNPK [45]. Lnc-FAM84B-4 interacts with HNRNPK, promoting cancer progression by suppressing the expression of the MAPK phosphatase DUSP1 [46]. In this study, LINC00571 was identified as a new binding partner of HNRNPK that interacts with the KH3 structural domain of HNRNPK. In addition, our results showed that the interaction between HNRNPK and ILF2 leads to a decrease in the ubiquitination of ILF2, thereby increasing its stability. Although previous research has shown that CRBN modulates the ubiquitination of ILF2 [47], our study significantly extends the current understanding of the regulatory mechanisms underlying ILF2 protein stability.

Although cancer cells have previously believed to mainly use glycolysis, the role of the TCA cycle in cancer metabolism and tumorigenesis has not been emphasized until recently [30]. IDH2 is a key enzyme in the TCA cycle that catalyzes the conversion of isocitrate to α-KG, a critical step in cellular metabolism and energy production [48]. Previous studies have demonstrated that Nrf2 and EZH2 regulate IDH2 transcription [49, 50]. In this study, we found that ILF2 functions as a transcriptional regulator to promote IDH2 transcription and the TCA cycle. However, the specific transcriptional binding sites of ILF2 require further investigation. Moreover, α-KG functions in regulating prosurvival signaling [51]. Our results suggest that upregulation of IDH2 may stimulate oxidative TCA cycling, increase α-KG production and promote TNBC progression. Thus, this study highlights that the TCA cycle has an important role in the progression of TNBC.

In the present study, we verified the protumor effects of the LINC00571/HNRNPK/ILF2 complex in both vitro and in vivo. However, the study has certain limitations. First, immune responses within the model are neglected as immunodeficient nude mice are used. Since we did not identify a potential mouse homologue of human LINC00571 (data not shown), we could not directly test the significance of the LINC00571 complex in the immune microenvironment of BALB/c mice. Second, we did not validate whether our models can be generalized or extended to other types of tumor cells.

## Conclusions

In conclusion, our study elucidated the potential mechanism of lncRNA action in the TCA cycle. These findings suggested that the LINC00571/HNRNPK/ILF2/IDH2 axis influences the progression of triple-negative breast cancer by regulating TCA metabolites. This discovery provides a new theoretical framework and potential targets for clinical treatment.

### Supplementary Information

**Additional file 1.** Prognostic analysis of lncRNAs in TCGA triple-negative breast cancer patients.

**Additional file 2.** Sequence, secondary structure and protein coding capacity of LINC00571. (A) Schematic representation of the genomic locus of LINC00571 in the human genome (chromosome 13). (B) The nucleotide sequence of LINC00571. (C) The secondary structure of LINC00571 from the AnnoLnc database (http://annolnc.cbi.pku.edu.cn/). (D) Putative ORFs in the LINC00571 sequence as predicted by the ORF Finder. (E) Protein coding potential of the LINC00571 sequence based on five different metrics. (F) Determination of LINC00571 nuclear and cytoplasmic distribution by qRT-PCR analysis in MCF7 cells, Cytoplasmic and nuclear controls were GAPDH and U6, respectively. (G) RNA-FISH assay revealing the cytoplasmic localization of LINC00571 within MCF7 cells. Positive controls for cytoplasm (18S) and nucleus (U6) were labeled with Cy3 (red), while the LINC00571 probe was labeled with FITC (green). Nuclei were counterstained with DAPI (blue). scale bar:10μm. Statistical analyses are depicted in bar graphs. Data are presented as mean ± SD from three independent experiments. Significance levels are denoted as * for p<0.05, ** for*p*<0.01, and *** for *p*<0.001, as determined by the t-test.

**Additional file 3.** LINC00571 regulates proliferation and apoptosis of BT-549 cells. (A) Expression of LINC00571 in TNBC cells transfected with shNC, shLINC00571#1, or shLINC00571#2 was quantified by qRT-PCR assay. (B) Expression of LINC00571 in TNBC cells transfected with vector or LINC00571 was measured by qRT-PCR assay. (C-E) Proliferation rate status of BT-549 cells was evaluated using CCK-8, colony formation, and EdU assays. Scale bar: 50μm. (F) Cell cycle analysis was conducted on BT-549 cells via flow cytometry after staining with propidium iodide (PI). (G) Apoptosis assessment in BT-549 cells was performed using a flow cytometry assay with AnnexinV and propidium iodide (PI) staining. Statistical analyses are depicted in bar graphs. Data are presented as mean ± SD from three independent experiments. Significance levels are denoted as * for *p*<0.05, ** for *p*<0.01, and *** for *p*<0.001, as determined by the t-test.

**Additional file 4.** LINC00571 promotes the progression of BT-549 cells by TCA signaling pathway. (A) Differential expression of genes enriched in the TCA cycle pathway based on GSEA analysis from TCGA data. (B) PCR analysis was performed to reveal the expression profile of corresponding genes in BT-549 cells with LINC00571 knockdown, *n* = 3. (C) Left, oxygen consumption rate (OCR) was analyzed in BT-549 cells with LINC00571 knockdown (*n* = 4). Right, basal respiration, ATP-coupled respiration and maximal respiration (*n*= 4). (D) Left, oxygen consumption rate (OCR) was analyzed in BT-549 cells with LINC00571 overexpression (*n* = 4). Right, basal respiration, ATP-coupled respiration and maximal respiration (*n*= 4). (E, F) Relative lactate level (E) and relative ATP level (F) in BT-549 cells with LINC00571 knockdown (left) or LINC00571 overexpression (right) were shown, *n* = 3. (G) Relative citrate level and relative α-KG level in BT-549 cells with LINC00571 knockdown (left) or LINC00571 overexpression (right) were shown, *n* = 3. Statistical analyses are depicted in bar graphs. Data are presented as mean ± SD. Significance levels are denoted as * for *p*<0.05, ** for *p*<0.01, and *** for *p*<0.001, as determined by the t-test.

**Additional file 5.** Expression and regulation of HNRNPK and ILF2 in TNBC cells. (A) Immunoblot analyses were performed for HNRNPK and ILF2 on biotin-labeled sense and antisense LINC00571 probe pull-down eluates from MDA-MB-231 and BT-549 cell lysates, with GAPDH as a loading control. (B) qRT-PCR assay depicted the expression level of HNRNPK (left) and ILF2 (right) in TNBC cells with LINC00571#1 knockdown. (C) Immunoblot (IB) analysis displayed the levels of HNRNPK and ILF2 in TNBC cells with LINC00571#1 knockdown. GAPDH was utilized as a loading control. (D) qRT-PCR assay illustrated the expression level of HNRNPK (left) and ILF2 (right) in TNBC cells with LINC00571 overexpression. (E) Immunoblot (IB) analysis showcased the levels of HNRNPK and ILF2 in TNBC cells with LINC00571 overexpression. GAPDH was utilized as a loading control. (F, G) Immunoblot (IB) analysis presented the levels of HNRNPK in TNBC cells with HNRNPK knockdown (F) or HNRNPK overexpression (G). GAPDH was utilized as a loading control. (H, I) Immunoblot (IB) analysis demonstrated the levels of ILF2 in TNBC cells with ILF2 knockdown (H) or ILF2 overexpression (I). GAPDH was utilized as a loading control. Statistical analyses are depicted in bar graphs. Data are presented as mean ± SD from three independent experiments. Significance levels are denoted as * for*p*<0.05, ** for *p*<0.01, and *** for *p*<0.001, as determined by the t-test.

**Additional file 6.** IDH2 expression and prognostic significance in breast cancer. (A-D) The expression of IDH2 in breast cancer was extracted from the UALCAN website utilizing the TCGA dataset. IDH2 expression was compared between normal breast tissue and primary breast tumors (A), among breast cancer subclasses (B), and across individual cancer stages (C). Additionally, IDH2 expression was assessed in relation to nodal metastasis status (D). (E-G) Kaplan-Meier Plotter database analysis was conducted to illustrate the impact of IDH2 expression on the overall survival (OS) (F), relapse-free survival (RFS) (G), and distant metastasis-free survival (DMFS) (H) of breast cancer patients in the Liu_2014 dataset. The patients were stratified based on high and low expression levels of IDH2.(TIF 912 KB)

**Additional file 7.** Knockdown of IDH2 rescues the phenotypes induced by ILF2 overexpression. (A-C) Proliferation rate status of BT-549 cells was assessed through CCK-8, colony formation assays, and EdU assays, *n* = 3. Scale bar: 50μm. (D) Cell cycle analysis was conducted via flow cytometry, involving propidium iodide (PI) staining on BT-549 cells, *n* = 3. (E) Apoptosis detection was performed using a flow cytometry assay. Annexin V and propidium iodide (PI) staining were employed on BT-549 cells, *n* = 3. (F) Left, oxygen consumption rate (OCR) on addition of oligomycin (Oligo), fluorocarbonyl cyanide phenylhydrazone (FCCP) and rotenone plus antimycin A (R&A) (*n* = 4). Right, basal respiration, ATP-coupled respiration and maximal respiration (*n*= 4). (G-H) Relative lactate level (G) and relative ATP level (H) in BT-549 cells co-transfected with ILF2 and shIDH2 were shown, *n* = 3. (I-J) Relative citrate level (I) and relative α-KG level (J) in BT-549 cells co-transfected with ILF2 and shIDH2 were shown, *n* = 3. Statistical analyses are depicted in bar graphs. Data are presented as mean ± SD. Significance levels are denoted as* for *p*<0.05, ** for p<0.01, and *** for *p*<0.001, as determined by the t-test.

**Additional file 8.** Knockdown of IDH2 rescues the effects induced by LINC00571 overexpression. (A-C) The proliferation rate of BT-549 cells was assessed through CCK-8, colony formation assays, and EdU assays. These assays were conducted on BT-549 cells co-transfected with LINC00571 and shIDH2. Scale bar: 50μm.(D) Cell cycle analysis was conducted using flow cytometry, and BT-549 cells were stained with propidium iodide (PI). (E) Apoptosis was detected using a flow cytometry assay. BT-549 cells were stained with Annexin V and propidium iodide (PI). Statistical analyses are depicted in bar graphs. Data are presented as mean ± SD from three independent experiments. Significance levels are denoted as * for *p*<0.05, ** for *p*<0.01, and *** for *p*<0.001, as determined by the t-test.

**Additional file 9.** LINC00571 mediates IDH2 expression to regulate the progression of DMA-MB-231 cells in vivo. (A-C) MDA-MB-231 cells, co-transfected with shLINC00571 and IDH2, were injected into the mammary fat pads of BALB/c nude mice. Images of tumors are shown (F) and eights (B) and tumor volume (C) were recorded, *n* = 5. Data are presented as mean ± SD. Significance levels are denoted as * for*p*<0.05, ** for *p*<0.01, and *** for *p*<0.001, as determined by the t-test.

**Additional file 10.** The supplementation of α-KG rescues the phenotypes induced by LINC00571 knockdown. (A-C) Proliferation status of MDA-MB-231 cells with LINC00571 knockdown and α-KG supplementation was assessed using CCK-8, colony formation assays, and EdU assays, *n* = 3. scale bar: 50μm. (D) Cell cycle analysis was conducted via flow cytometry, and MDA-MB-231 cells were stained with propidium iodide (PI), *n* = 3. (E) Apoptosis was detected using a flow cytometry assay, with Annexin V and propidium iodide (PI) staining in MDA-MB-231 cells, *n* = 3. (F-J) In vivo studies involved subcutaneous injection of MDA-MB-231 cells with LINC00571 knockdown and α-KG supplementation into BALB/c athymic nude mice, *n* = 5. Images of xenograft tumors were captured using a digital camera (F). Tumor weight was measured on day 50 (G). Tumor volume was monitored every ten days, calculated using the formula: volume = length × (width)²/2 (H). Immunohistochemical images of Ki67 and PCNA staining were conducted for the control, shLINC00571, α-KG, and shLINC00571 with α-KG groups. scale bar: 100μm. (I). Immunofluorescence images illustrated TUNEL staining in the control, shLINC00571-, α-KG-, and shLINC00571 combined plus α-KG-treated groups. scale bar: 60μm. (J). Statistical analyses are depicted in bar graphs. Data are presented as mean ± SD. Significance levels are denoted as * for *p*<0.05, ** for *p*<0.01, and *** for *p*<0.001, as determined by the t-test.

**Additional file 11.** Replicate data for cell cycle. (A-B) Replicate data of Fig. 2D. (C) Replicate data of Fig. 7D. (D) Replicate data of Fig. 8D. (E-F) Replicate data of Additional file 3F. (G) Replicate data of Additional file 7D. (H) Replicate data of Additional file 8D. (I) Replicate data of Additional file 9D.

**Additional file 12.** Replicate data for apoptosis. (A-B) Replicate data of Fig. 2D. (C) Replicate data of Fig. 7D. (D) Replicate data of Fig. 8D. (E-F) Replicate data of Additional file 3F. (G) Replicate data of Additional file 7D. (H) Replicate data of Additional file 8D. (I) Replicate data of Additional file 9D.

**Additional file 13.** The sequences of primers, oligonucleotides and probes used in this study.

## Data Availability

The datasets used and/or analysed during the current study are available from the corresponding author on reasonable request.

## Abbreviations

TNBC: Triple-negative breast cancer
lncRNAs: Long non-coding RNAs
RBPs: RNA binding proteins
HNRNPK: Heterogeneous nuclear ribonucleoprotein K
TCA: Tricarboxylic acid
FH: Fumarase
KGDHC: α-ketoglutarate dehydrogenase complex
ATCC: American Type Culture Collection
FISH: fluorescence in situ hybridization
CCK-8: Cell Counting Kit-8
OCR: Oxygen consumption rates
FCCP: Carbonyl cyanide-4-(trifluoromethoxy) phenylhydrazone
RIP: RNA immunoprecipitation
TCGA: The Cancer Genome Atlas
MFE: Minimum free energy
GSEA: Gene set enrichment analysis
GLS: Glutaminase
GLUD: Glutamate dehydrogenase
α-KG: α-ketoglutarate
KH: k-homology
CHX: Cycloheximide
OS: Overall survival
RFS: Recurrence free survival
DMFS: Distant metastasis-free survival
