# Supplementary figures and images for: LINC00571 drives tricarboxylic acid cycle metabolism in triple-negative breast cancer through HNRNPK/ILF2/IDH2 axis
Source: J Exp Clin Cancer Res. 2024 Jan 18;43:22. doi: 10.1186/s13046-024-02950-y (PMC10795234; doi:10.1186/s13046-024-02950-y)

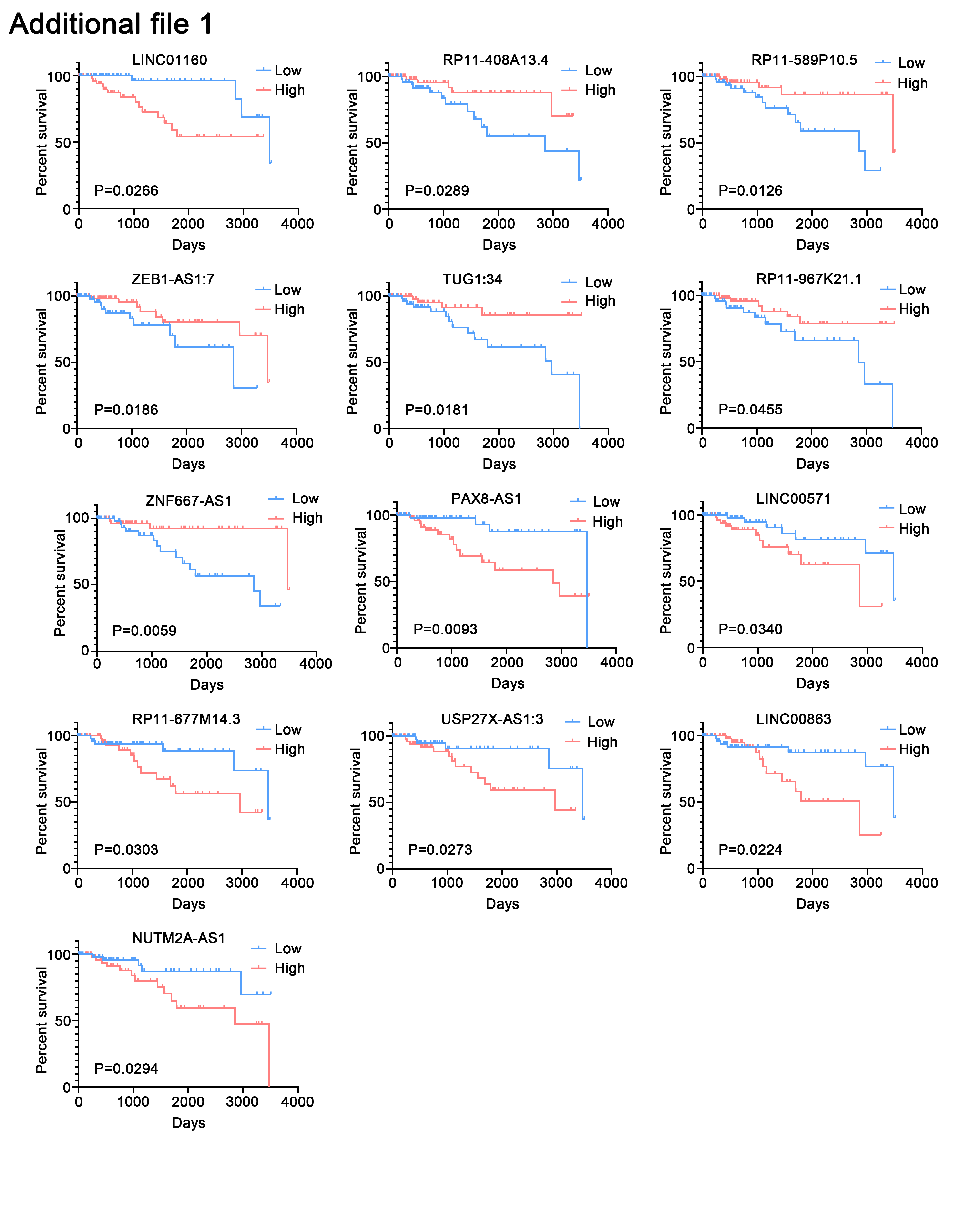

Supplement: Supplementary file 1 — Additional file 1. Prognostic analysis of lncRNAs in TCGA triple-negative breast cancer patients. [file 13046_2024_2950_MOESM1_ESM.tif]

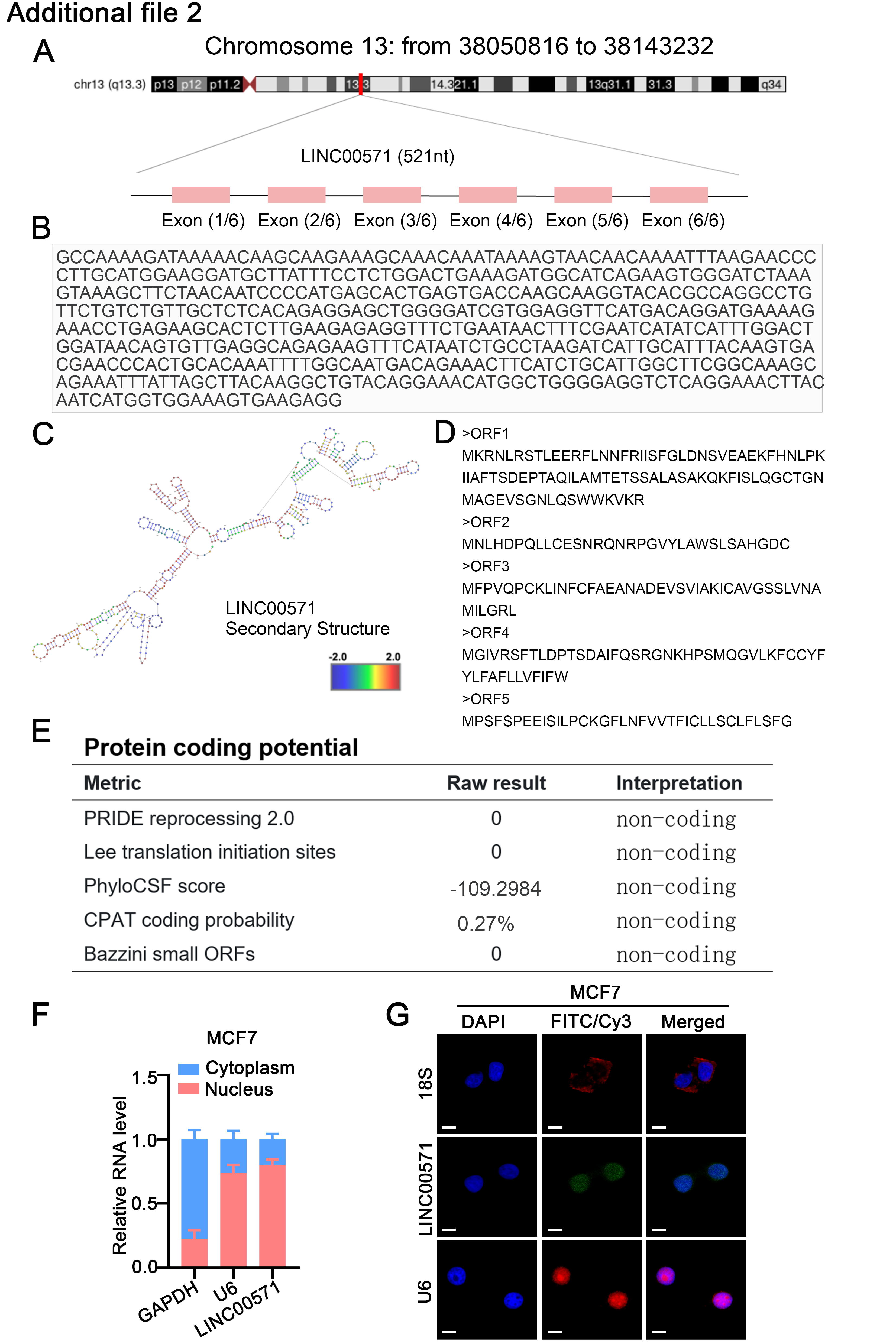

Supplement: Supplementary file 2 — Additional file 2. Sequence, secondary structure and protein coding capacity of LINC00571. (A) Schematic representation of the genomic locus of LINC00571 in the human genome (chromosome 13). (B) The nucleotide sequence of LINC00571. (C) The secondary structure of LINC00571 from the AnnoLnc database (http://annolnc.cbi.pku.edu.cn/). (D) Putative ORFs in the LINC00571 sequence as predicted by the ORF Finder. (E) Protein coding potential of the LINC00571 sequence based on five different metrics. (F) Determination of LINC00571 nuclear and cytoplasmic distribution by qRT-PCR analysis in MCF7 cells, Cytoplasmic and nuclear controls were GAPDH and U6, respectively. (G) RNA-FISH assay revealing the cytoplasmic localization of LINC00571 within MCF7 cells. Positive controls for cytoplasm (18S) and nucleus (U6) were labeled with Cy3 (red), while the LINC00571 probe was labeled with FITC (green). Nuclei were counterstained with DAPI (blue). scale bar:10μm. Statistical analyses are depicted in bar graphs. Data are presented as mean ± SD from three independent experiments. Significance levels are denoted as * for p<0.05, ** forp<0.01, and *** for p<0.001, as determined by the t-test. [file 13046_2024_2950_MOESM2_ESM.tif]

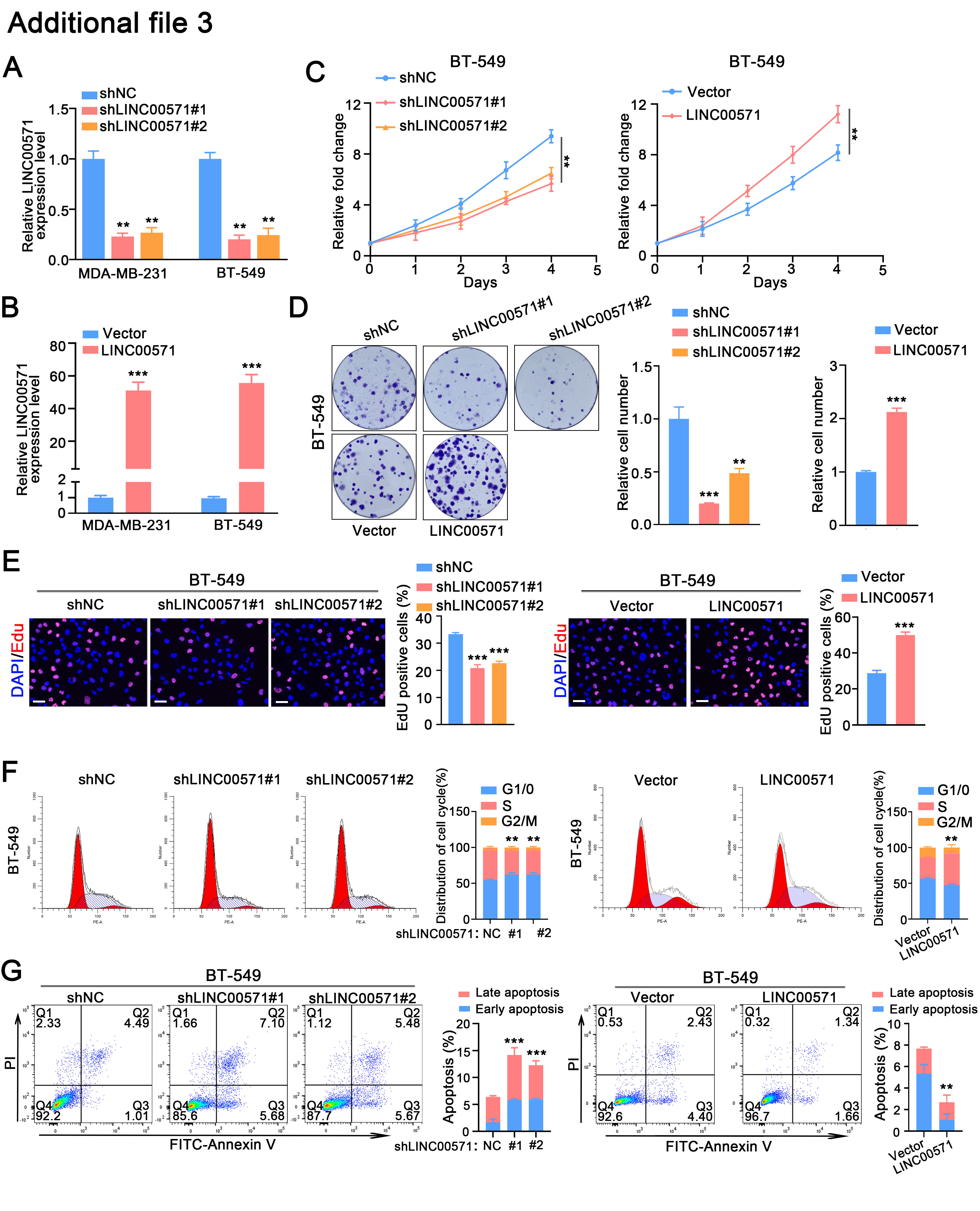

Supplement: Supplementary file 3 — Additional file 3. LINC00571 regulates proliferation and apoptosis of BT-549 cells. (A) Expression of LINC00571 in TNBC cells transfected with shNC, shLINC00571#1, or shLINC00571#2 was quantified by qRT-PCR assay. (B) Expression of LINC00571 in TNBC cells transfected with vector or LINC00571 was measured by qRT-PCR assay. (C-E) Proliferation rate status of BT-549 cells was evaluated using CCK-8, colony formation, and EdU assays. Scale bar: 50μm. (F) Cell cycle analysis was conducted on BT-549 cells via flow cytometry after staining with propidium iodide (PI). (G) Apoptosis assessment in BT-549 cells was performed using a flow cytometry assay with AnnexinV and propidium iodide (PI) staining. Statistical analyses are depicted in bar graphs. Data are presented as mean ± SD from three independent experiments. Significance levels are denoted as * for p<0.05, ** for p<0.01, and *** for p<0.001, as determined by the t-test. [file 13046_2024_2950_MOESM3_ESM.tif]

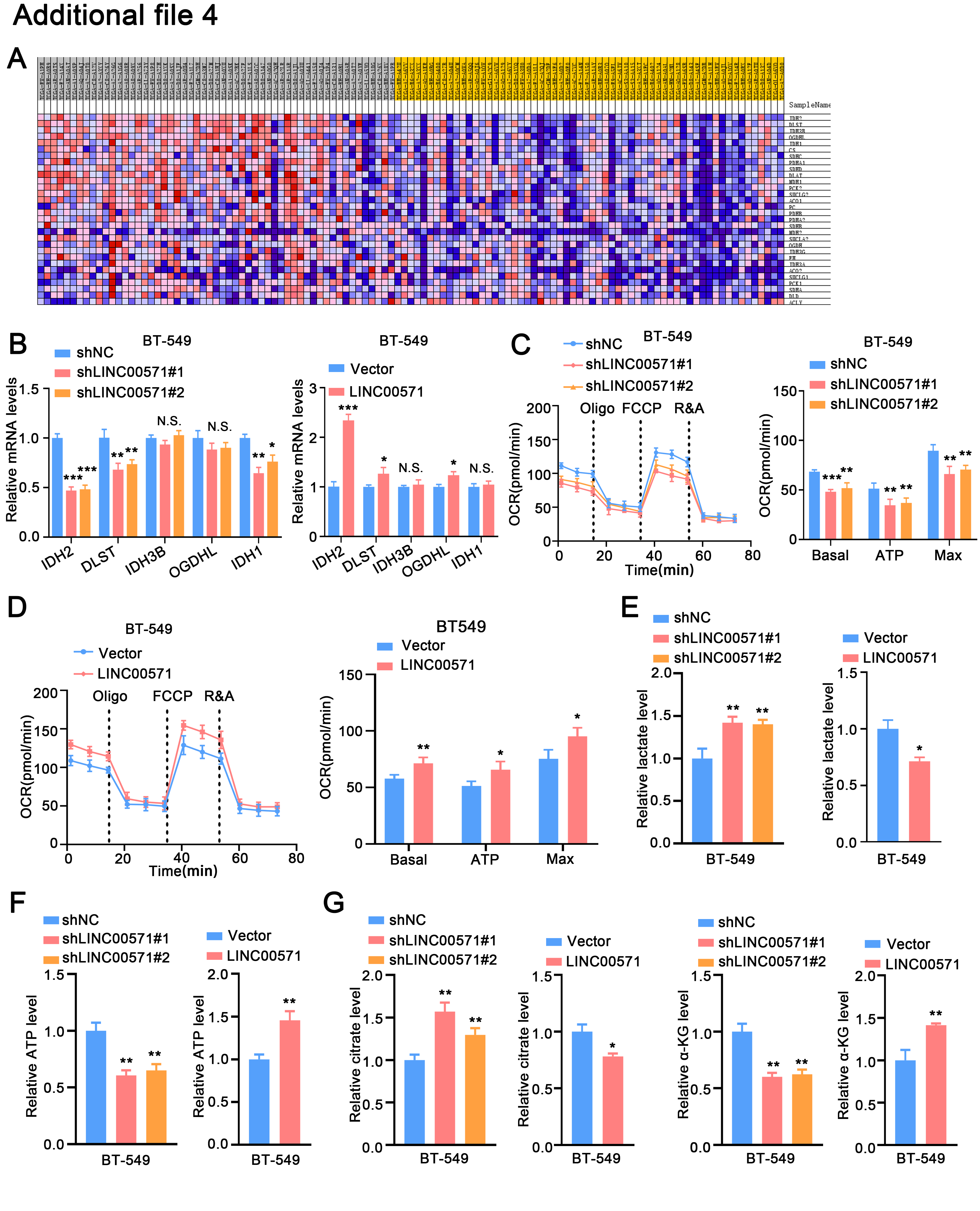

Supplement: Supplementary file 4 — Additional file 4. LINC00571 promotes the progression of BT-549 cells by TCA signaling pathway. (A) Differential expression of genes enriched in the TCA cycle pathway based on GSEA analysis from TCGA data. (B) PCR analysis was performed to reveal the expression profile of corresponding genes in BT-549 cells with LINC00571 knockdown, n = 3. (C) Left, oxygen consumption rate (OCR) was analyzed in BT-549 cells with LINC00571 knockdown (n = 4). Right, basal respiration, ATP-coupled respiration and maximal respiration (n= 4). (D) Left, oxygen consumption rate (OCR) was analyzed in BT-549 cells with LINC00571 overexpression (n = 4). Right, basal respiration, ATP-coupled respiration and maximal respiration (n= 4). (E, F) Relative lactate level (E) and relative ATP level (F) in BT-549 cells with LINC00571 knockdown (left) or LINC00571 overexpression (right) were shown, n = 3. (G) Relative citrate level and relative α-KG level in BT-549 cells with LINC00571 knockdown (left) or LINC00571 overexpression (right) were shown, n = 3. Statistical analyses are depicted in bar graphs. Data are presented as mean ± SD. Significance levels are denoted as * for p<0.05, ** for p<0.01, and *** for p<0.001, as determined by the t-test. [file 13046_2024_2950_MOESM4_ESM.tif]

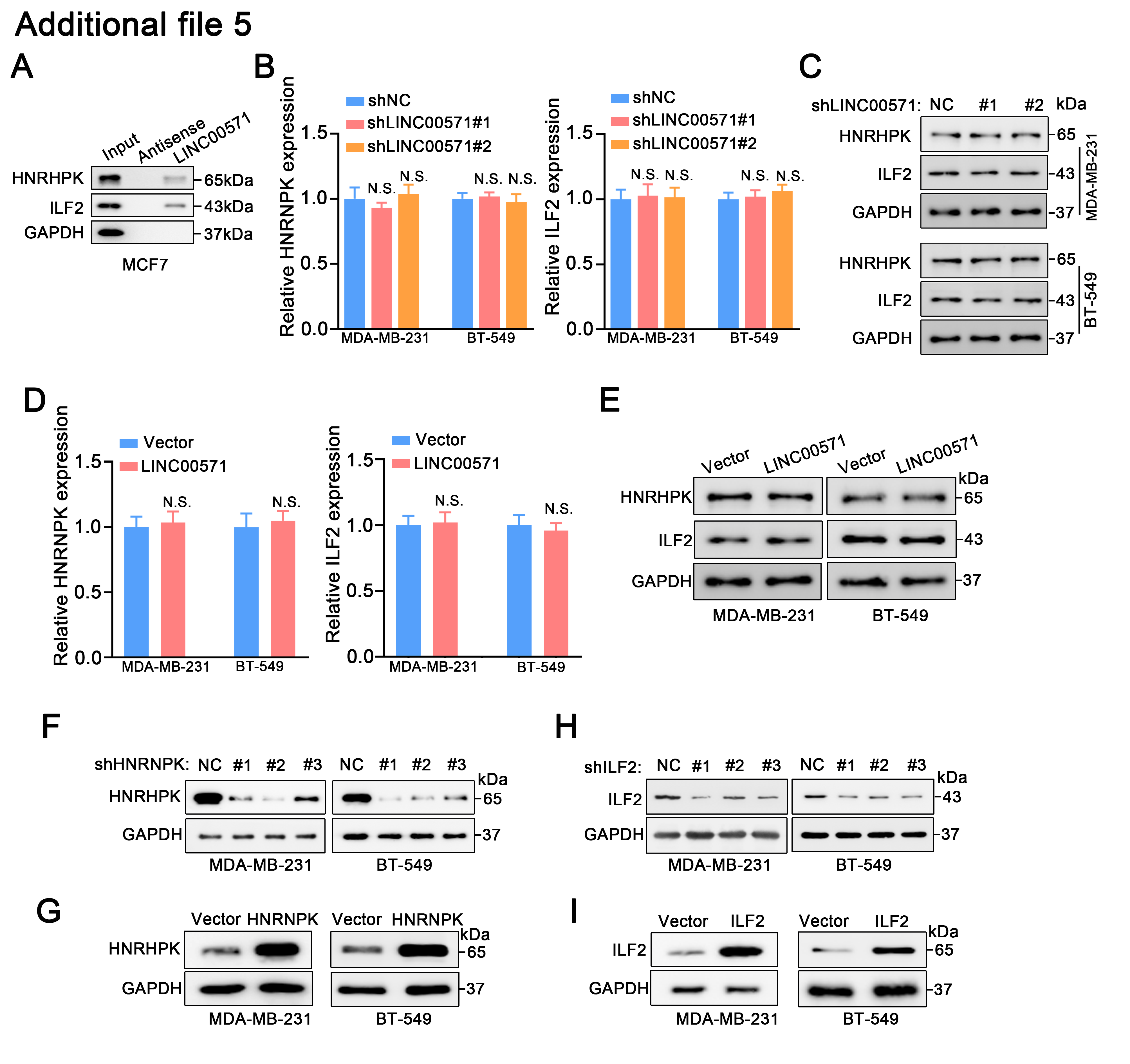

Supplement: Supplementary file 5 — Additional file 5. Expression and regulation of HNRNPK and ILF2 in TNBC cells. (A) Immunoblot analyses were performed for HNRNPK and ILF2 on biotin-labeled sense and antisense LINC00571 probe pull-down eluates from MDA-MB-231 and BT-549 cell lysates, with GAPDH as a loading control. (B) qRT-PCR assay depicted the expression level of HNRNPK (left) and ILF2 (right) in TNBC cells with LINC00571#1 knockdown. (C) Immunoblot (IB) analysis displayed the levels of HNRNPK and ILF2 in TNBC cells with LINC00571#1 knockdown. GAPDH was utilized as a loading control. (D) qRT-PCR assay illustrated the expression level of HNRNPK (left) and ILF2 (right) in TNBC cells with LINC00571 overexpression. (E) Immunoblot (IB) analysis showcased the levels of HNRNPK and ILF2 in TNBC cells with LINC00571 overexpression. GAPDH was utilized as a loading control. (F, G) Immunoblot (IB) analysis presented the levels of HNRNPK in TNBC cells with HNRNPK knockdown (F) or HNRNPK overexpression (G). GAPDH was utilized as a loading control. (H, I) Immunoblot (IB) analysis demonstrated the levels of ILF2 in TNBC cells with ILF2 knockdown (H) or ILF2 overexpression (I). GAPDH was utilized as a loading control. Statistical analyses are depicted in bar graphs. Data are presented as mean ± SD from three independent experiments. Significance levels are denoted as * forp<0.05, ** for p<0.01, and *** for p<0.001, as determined by the t-test. [file 13046_2024_2950_MOESM5_ESM.tif]

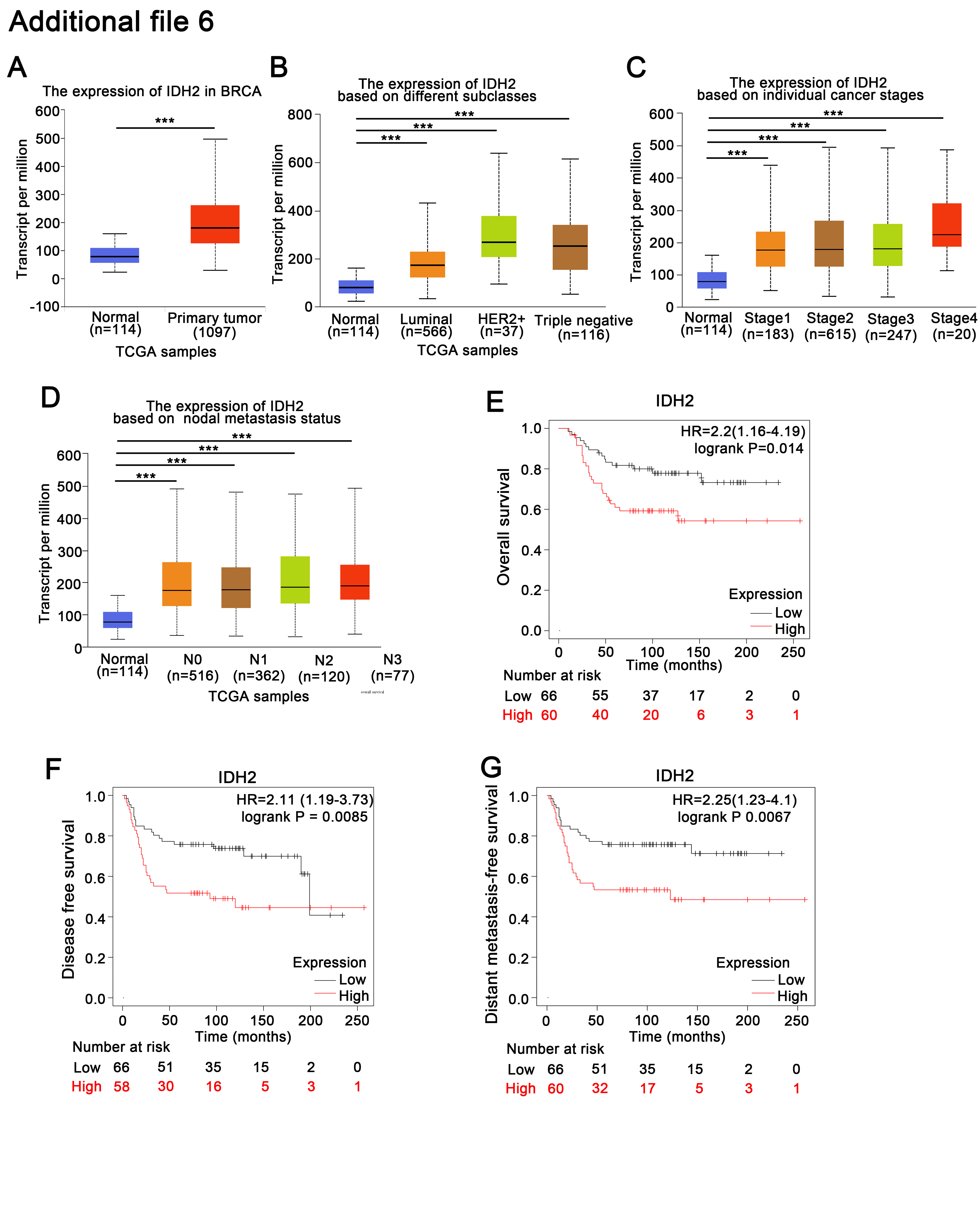

Supplement: Supplementary file 6 — Additional file 6. IDH2 expression and prognostic significance in breast cancer. (A-D) The expression of IDH2 in breast cancer was extracted from the UALCAN website utilizing the TCGA dataset. IDH2 expression was compared between normal breast tissue and primary breast tumors (A), among breast cancer subclasses (B), and across individual cancer stages (C). Additionally, IDH2 expression was assessed in relation to nodal metastasis status (D). (E-G) Kaplan-Meier Plotter database analysis was conducted to illustrate the impact of IDH2 expression on the overall survival (OS) (F), relapse-free survival (RFS) (G), and distant metastasis-free survival (DMFS) (H) of breast cancer patients in the Liu_2014 dataset. The patients were stratified based on high and low expression levels of IDH2.(TIF 912 KB) [file 13046_2024_2950_MOESM6_ESM.tif]

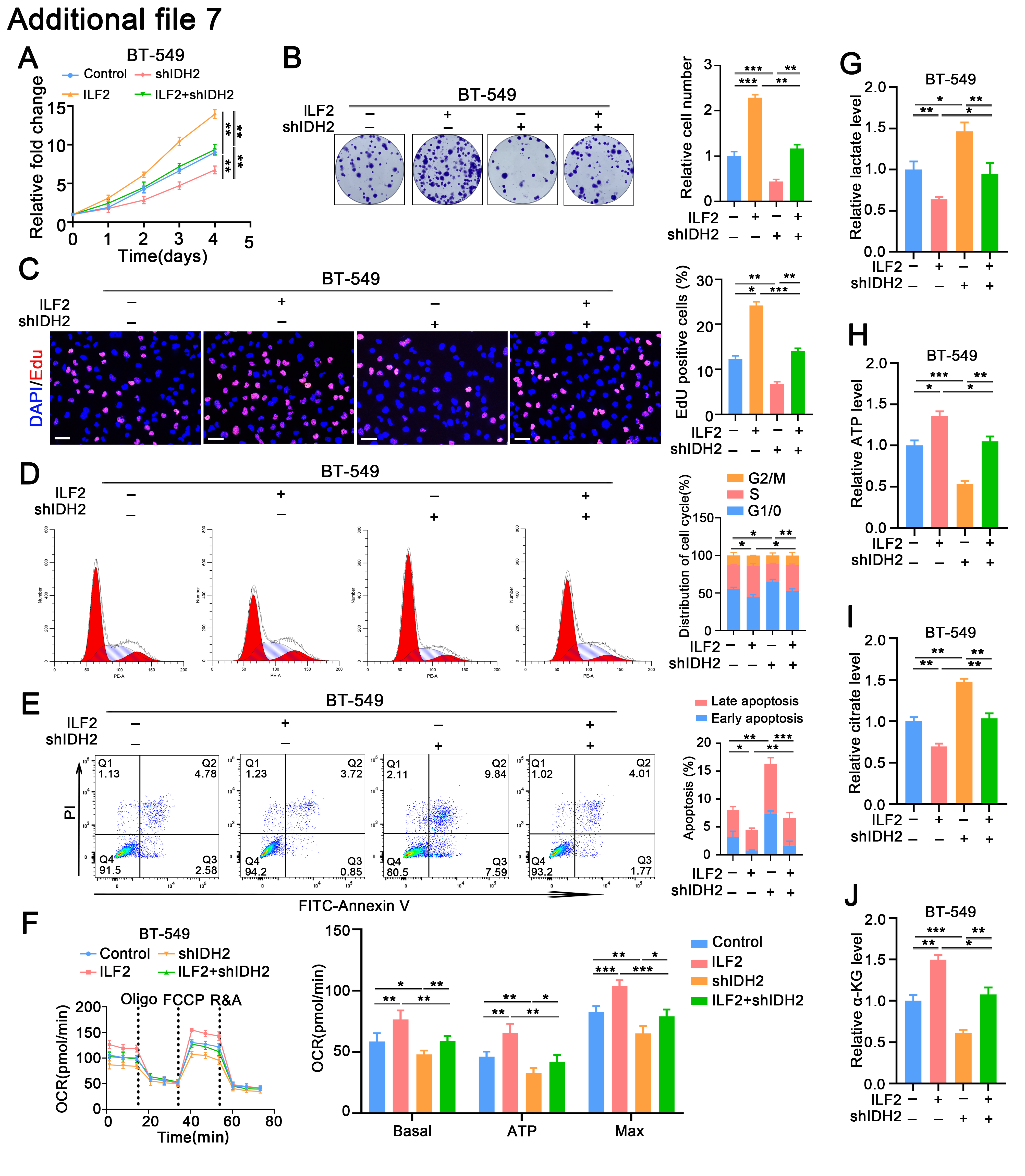

Supplement: Supplementary file 7 — Additional file 7. Knockdown of IDH2 rescues the phenotypes induced by ILF2 overexpression. (A-C) Proliferation rate status of BT-549 cells was assessed through CCK-8, colony formation assays, and EdU assays, n = 3. Scale bar: 50μm. (D) Cell cycle analysis was conducted via flow cytometry, involving propidium iodide (PI) staining on BT-549 cells, n = 3. (E) Apoptosis detection was performed using a flow cytometry assay. Annexin V and propidium iodide (PI) staining were employed on BT-549 cells, n = 3. (F) Left, oxygen consumption rate (OCR) on addition of oligomycin (Oligo), fluorocarbonyl cyanide phenylhydrazone (FCCP) and rotenone plus antimycin A (R&A) (n = 4). Right, basal respiration, ATP-coupled respiration and maximal respiration (n= 4). (G-H) Relative lactate level (G) and relative ATP level (H) in BT-549 cells co-transfected with ILF2 and shIDH2 were shown, n = 3. (I-J) Relative citrate level (I) and relative α-KG level (J) in BT-549 cells co-transfected with ILF2 and shIDH2 were shown, n = 3. Statistical analyses are depicted in bar graphs. Data are presented as mean ± SD. Significance levels are denoted as* for p<0.05, ** for p<0.01, and *** for p<0.001, as determined by the t-test. [file 13046_2024_2950_MOESM7_ESM.tif]

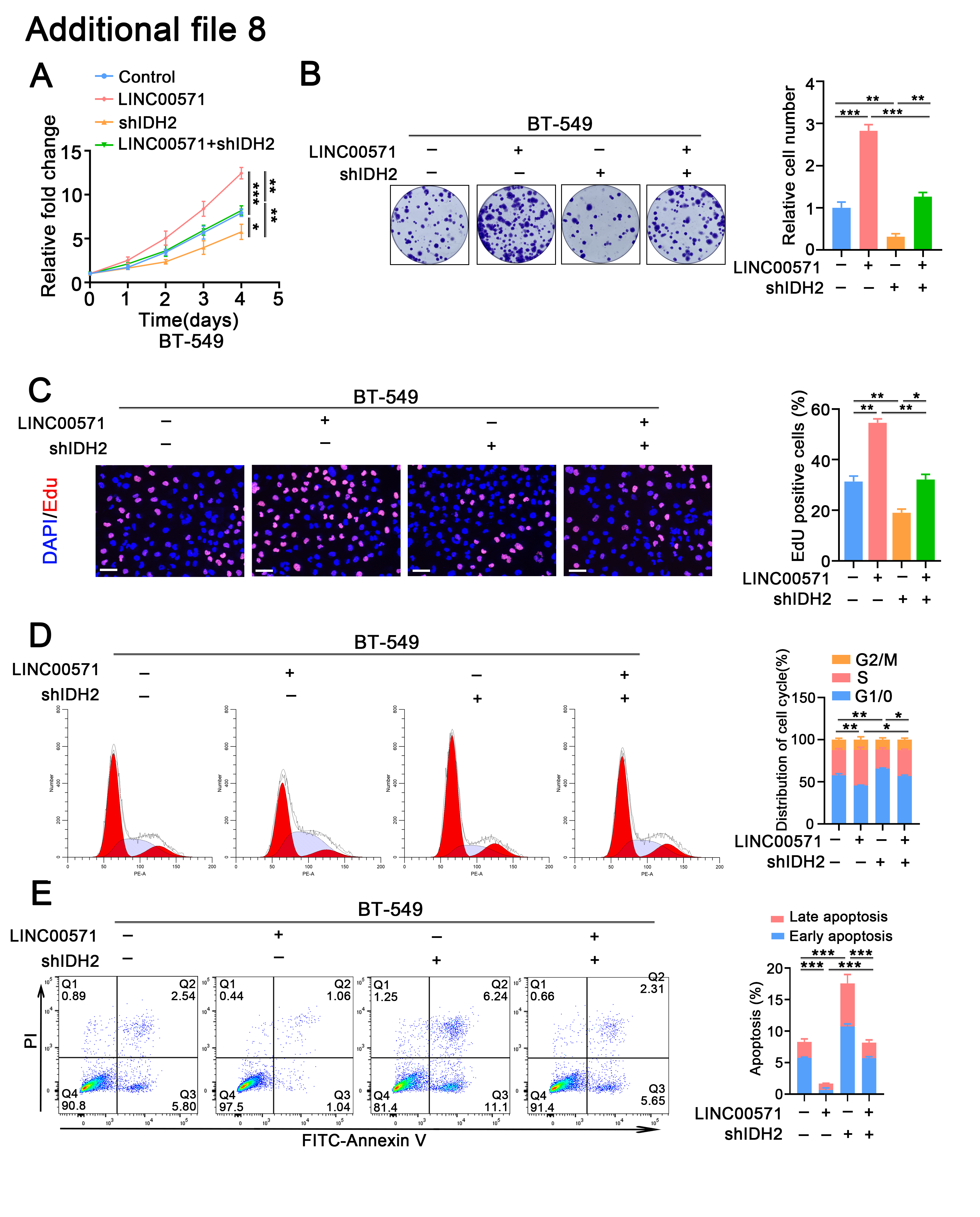

Supplement: Supplementary file 8 — Additional file 8. Knockdown of IDH2 rescues the effects induced by LINC00571 overexpression. (A-C) The proliferation rate of BT-549 cells was assessed through CCK-8, colony formation assays, and EdU assays. These assays were conducted on BT-549 cells co-transfected with LINC00571 and shIDH2. Scale bar: 50μm.(D) Cell cycle analysis was conducted using flow cytometry, and BT-549 cells were stained with propidium iodide (PI). (E) Apoptosis was detected using a flow cytometry assay. BT-549 cells were stained with Annexin V and propidium iodide (PI). Statistical analyses are depicted in bar graphs. Data are presented as mean ± SD from three independent experiments. Significance levels are denoted as * for p<0.05, ** for p<0.01, and *** for p<0.001, as determined by the t-test. [file 13046_2024_2950_MOESM8_ESM.tif]

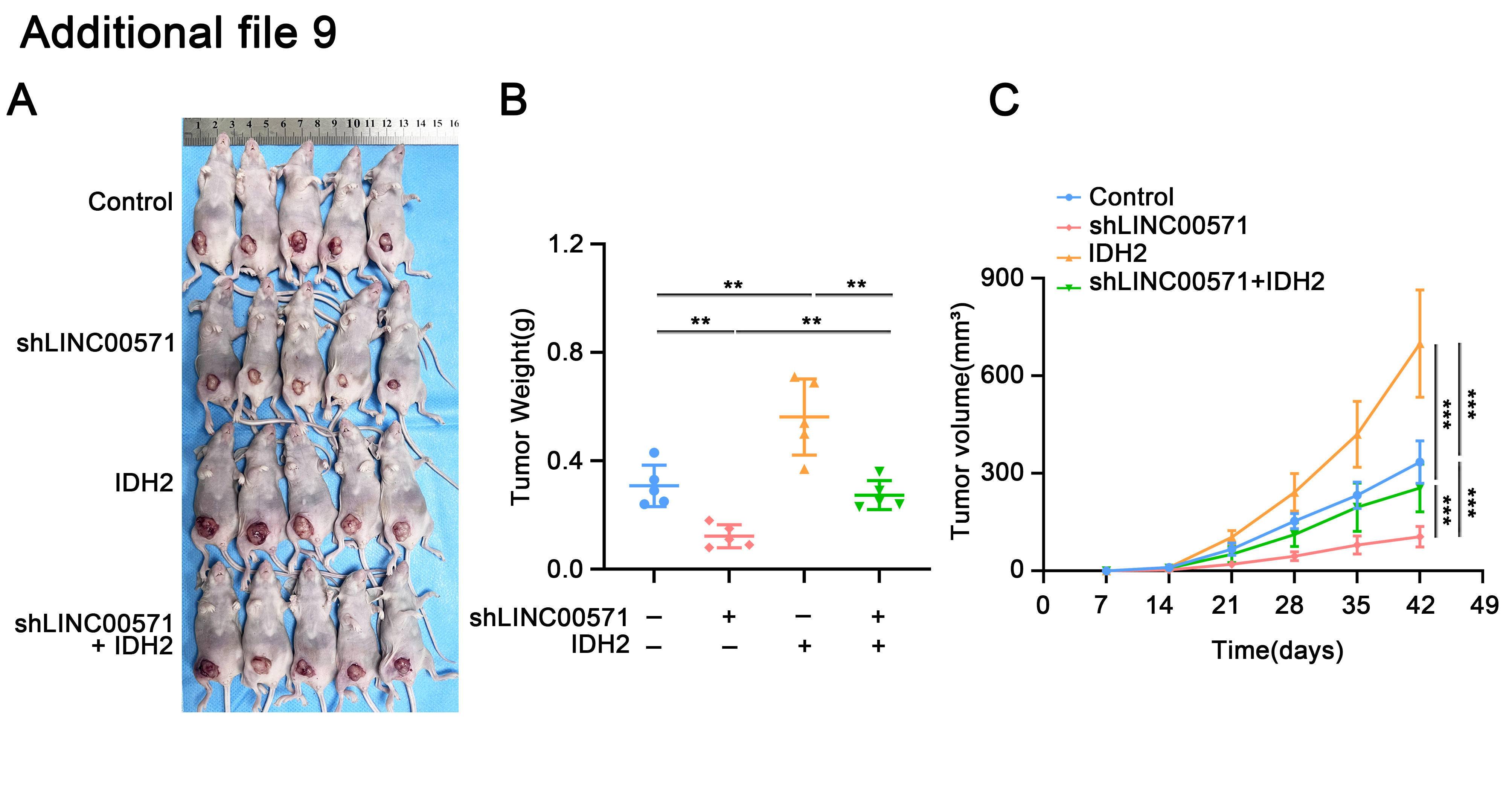

Supplement: Supplementary file 9 — Additional file 9. LINC00571 mediates IDH2 expression to regulate the progression of DMA-MB-231 cells in vivo. (A-C) MDA-MB-231 cells, co-transfected with shLINC00571 and IDH2, were injected into the mammary fat pads of BALB/c nude mice. Images of tumors are shown (F) and eights (B) and tumor volume (C) were recorded, n = 5. Data are presented as mean ± SD. Significance levels are denoted as * forp<0.05, ** for p<0.01, and *** for p<0.001, as determined by the t-test. [file 13046_2024_2950_MOESM9_ESM.tif]

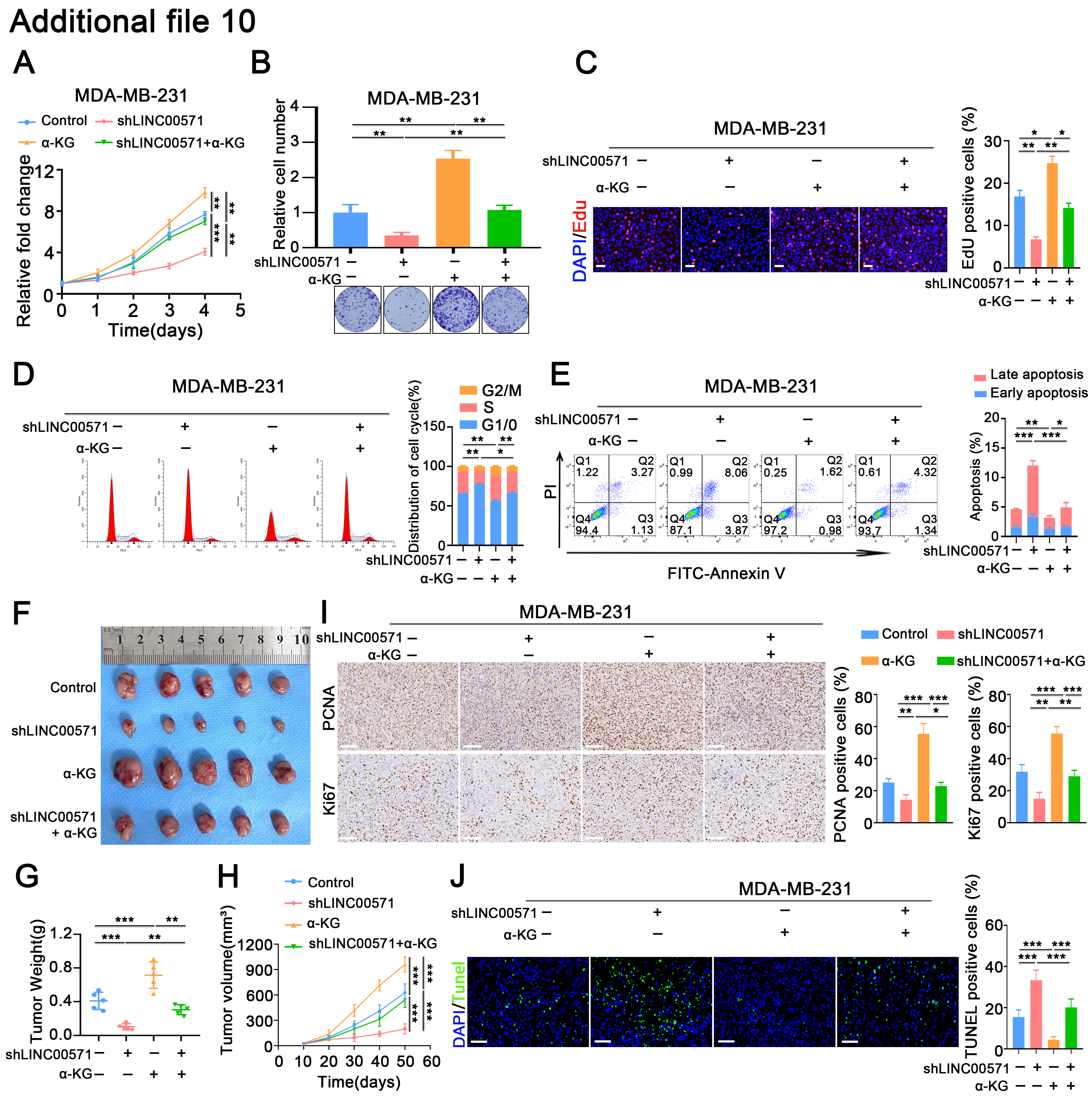

Supplement: Supplementary file 10 — Additional file 10. The supplementation of α-KG rescues the phenotypes induced by LINC00571 knockdown. (A-C) Proliferation status of MDA-MB-231 cells with LINC00571 knockdown and α-KG supplementation was assessed using CCK-8, colony formation assays, and EdU assays, n = 3. scale bar: 50μm. (D) Cell cycle analysis was conducted via flow cytometry, and MDA-MB-231 cells were stained with propidium iodide (PI), n = 3. (E) Apoptosis was detected using a flow cytometry assay, with Annexin V and propidium iodide (PI) staining in MDA-MB-231 cells, n = 3. (F-J) In vivo studies involved subcutaneous injection of MDA-MB-231 cells with LINC00571 knockdown and α-KG supplementation into BALB/c athymic nude mice, n = 5. Images of xenograft tumors were captured using a digital camera (F). Tumor weight was measured on day 50 (G). Tumor volume was monitored every ten days, calculated using the formula: volume = length × (width)²/2 (H). Immunohistochemical images of Ki67 and PCNA staining were conducted for the control, shLINC00571, α-KG, and shLINC00571 with α-KG groups. scale bar: 100μm. (I). Immunofluorescence images illustrated TUNEL staining in the control, shLINC00571-, α-KG-, and shLINC00571 combined plus α-KG-treated groups. scale bar: 60μm. (J). Statistical analyses are depicted in bar graphs. Data are presented as mean ± SD. Significance levels are denoted as * for p<0.05, ** for p<0.01, and *** for p<0.001, as determined by the t-test. [file 13046_2024_2950_MOESM10_ESM.tif]

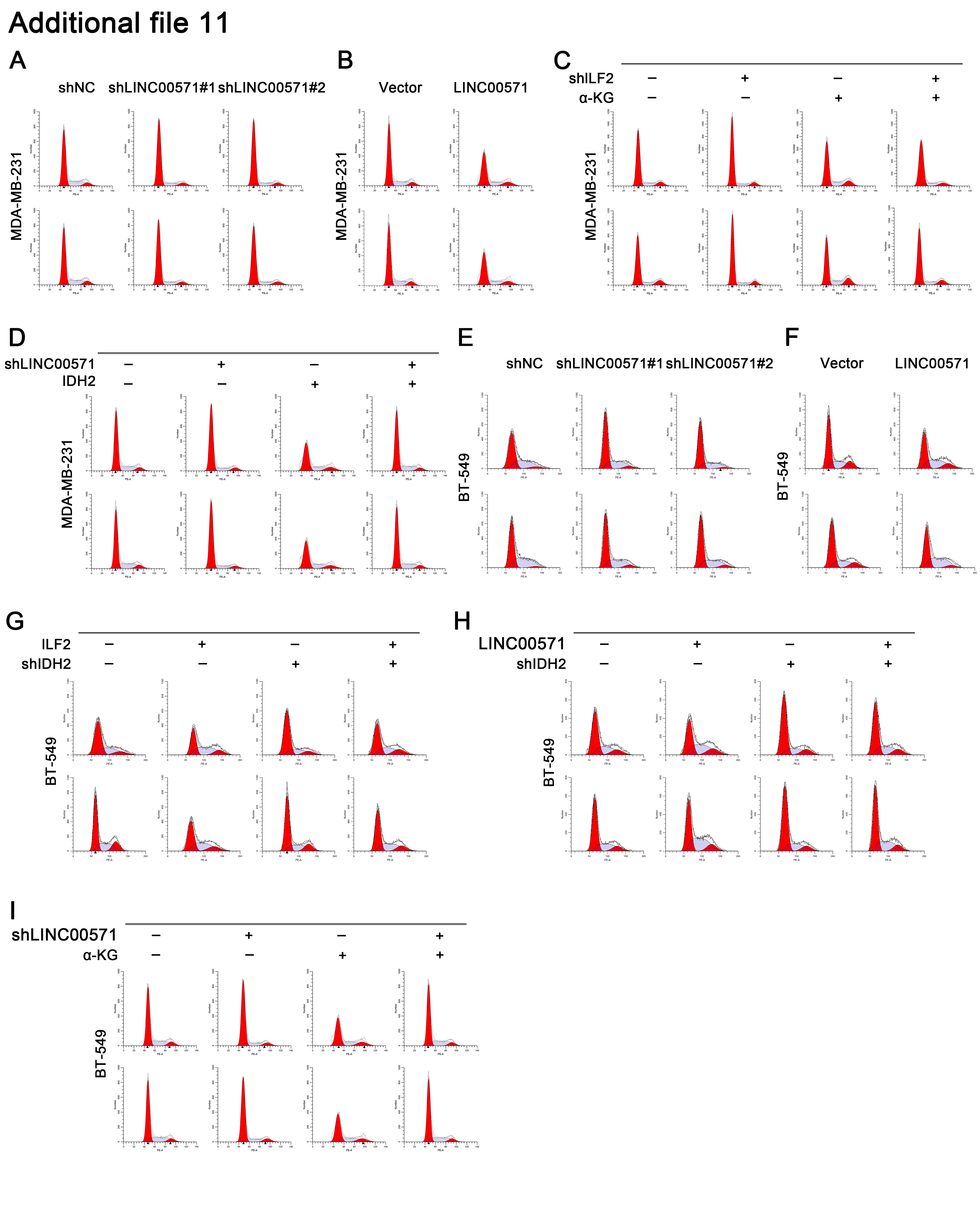

Supplement: Supplementary file 11 — Additional file 11. Replicate data for cell cycle. (A-B) Replicate data of Fig. 2D. (C) Replicate data of Fig. 7D. (D) Replicate data of Fig. 8D. (E-F) Replicate data of Additional file 3F. (G) Replicate data of Additional file 7D. (H) Replicate data of Additional file 8D. (I) Replicate data of Additional file 9D. [file 13046_2024_2950_MOESM11_ESM.tif]

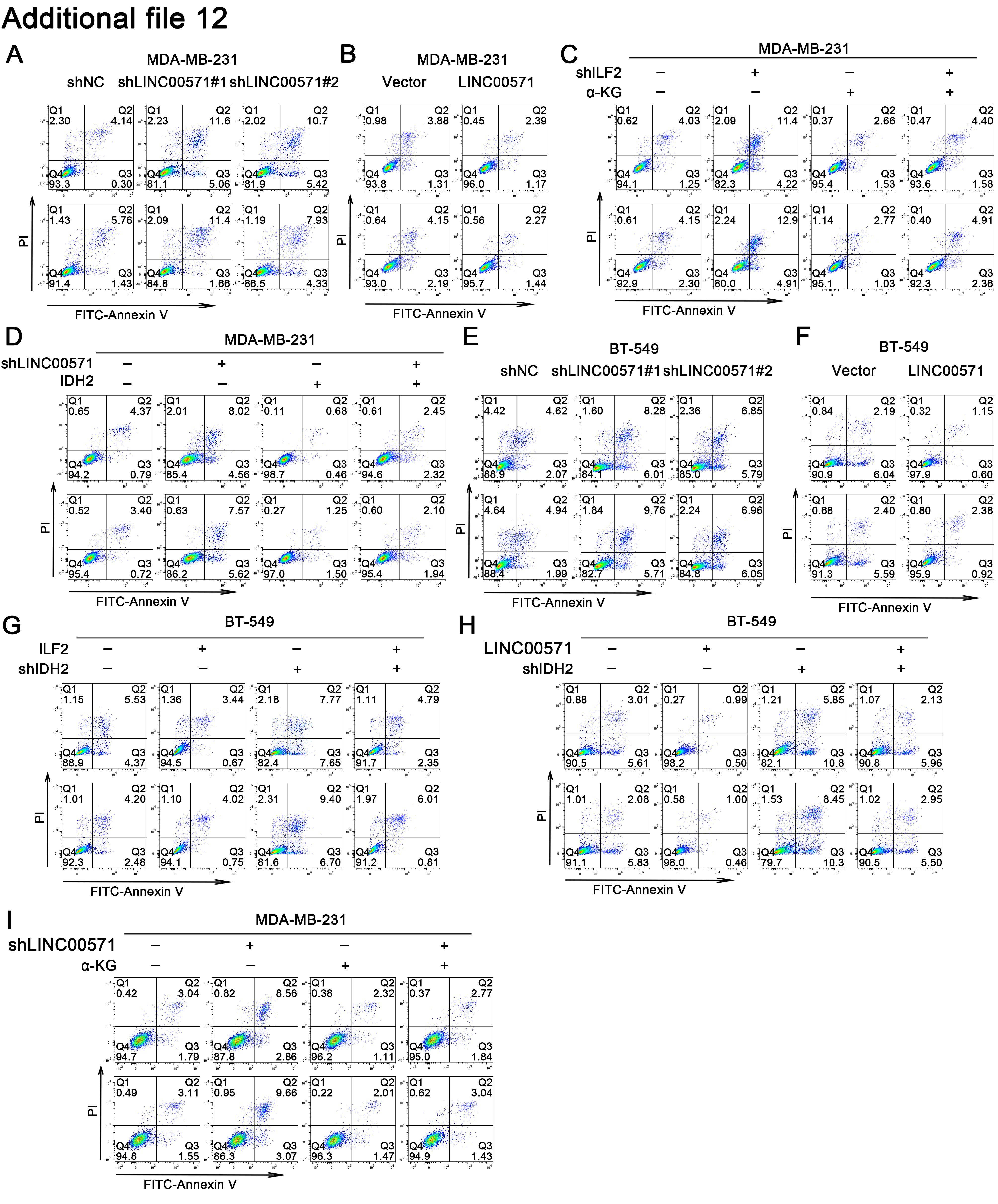

Supplement: Supplementary file 12 — Additional file 12. Replicate data for apoptosis. (A-B) Replicate data of Fig. 2D. (C) Replicate data of Fig. 7D. (D) Replicate data of Fig. 8D. (E-F) Replicate data of Additional file 3F. (G) Replicate data of Additional file 7D. (H) Replicate data of Additional file 8D. (I) Replicate data of Additional file 9D. [file 13046_2024_2950_MOESM12_ESM.tif]
